# Temperature response of wheat affects final height and the timing of stem elongation under field conditions

**DOI:** 10.1093/jxb/eraa471

**Published:** 2020-10-15

**Authors:** Lukas Kronenberg, Steven Yates, Martin P Boer, Norbert Kirchgessner, Achim Walter, Andreas Hund

**Affiliations:** 1 Crop Science, Institute of Agricultural Sciences, ETH Zürich, Zurich, Switzerland; 2 Molecular Plant Breeding, Institute of Agricultural Sciences, ETH Zürich, Zurich, Switzerland; 3 Biometris, Wageningen University & Research, PB Wageningen, The Netherlands; 4 John Innes Centre, UK

**Keywords:** Development, field phenotyping, GWAS, LIDAR, physiology, plant height, temperature response, wheat

## Abstract

In wheat, temperature affects the timing and intensity of stem elongation. Genetic variation for this process is therefore important for adaptation. This study investigates the genetic response to temperature fluctuations during stem elongation and its relationship to phenology and height. Canopy height of 315 wheat genotypes (GABI wheat panel) was scanned twice weekly in the field phenotyping platform (FIP) of ETH Zurich using a LIDAR. Temperature response was modelled using linear regressions between stem elongation and mean temperature in each measurement interval. This led to a temperature-responsive (slope) and a temperature-irresponsive (intercept) component. The temperature response was highly heritable (*H*^2^=0.81) and positively related to a later start and end of stem elongation as well as final height. Genome-wide association mapping revealed three temperature-responsive and four temperature-irresponsive quantitative trait loci (QTLs). Furthermore, putative candidate genes for temperature-responsive QTLs were frequently related to the flowering pathway in *Arabidopsis thaliana*, whereas temperature-irresponsive QTLs corresponded to growth and reduced height genes. In combination with *Rht* and *Ppd* alleles, these loci, together with the loci for the timing of stem elongation, accounted for 71% of the variability in height. This demonstrates how high-throughput field phenotyping combined with environmental covariates can contribute to a smarter selection of climate-resilient crops.

## Teaser

Temperature response of stem elongation in wheat grown under field conditions indicated that temperature response is highly heritable and linked to the flowering pathway.

## Introduction

Temperature is a major abiotic factor affecting plant growth and development. As a consequence of global warming, wheat production could decrease by 6% for each degree Celsius of global temperature increase (Asseng *et al.*, 2015). While heat stress during critical stages can drastically reduce yield (Gibson and Paulsen, 1999; Farooq *et al.*, 2011), warm temperatures can decrease yield by accelerating development and thereby shortening critical periods for yield formation (Fischer, 1985; Slafer and Rawson, 1994). Despite the clear effect of temperature on growth and phenology, little is known about the genotype-specific response pattern to varying temperature conditions during crop development and its genetic control. We therefore aimed to quantify the genotype-specific temperature responsiveness of European winter wheat during the stem elongation (SE) phase.

SE is a critical phase for yield formation in wheat. It occurs between the phenological stages of terminal spikelet initiation and anthesis (Slafer *et al.*, 2015). The start of SE coincides with the transition from vegetative to reproductive development, when the apex meristem differentiates from producing leaf primordia to producing spikelet primordia (Trevaskis *et al.*, 2007; Kamran *et al.*, 2014). During SE, florets are initiated at the spikelets until booting (Kirby, 1988; Slafer *et al.*, 2015). An increased duration of SE increases the number of fertile florets due to longer spike growth and higher dry matter partitioning to the spike (González *et al.*, 2003). This in turn increases the number of grains per spike and therefore yield (Fischer, 1985). Modifying the timing of the critical phenological stages (transition to early reproductive phase and flowering), and thus SE duration, has been proposed as a way to increase wheat yield (Slafer *et al.*, 1996; Miralles and Slafer, 2007; Whitechurch *et al.*, 2007) or at least to mitigate adverse climate change effects on yield, for example by enhancing earliness to escape heat during flowering (Chapman *et al.*, 2012; Hernandez-Ochoa *et al.*, 2019). The recent warming trend causes a faster advancement in phenology. For example, flowering time occurred earlier in Germany throughout the past decade, which is attributable to both increased temperature and selection for early flowering (Rezaei *et al.*, 2018).

Final height is also an important yield determinant. During the ‘Green Revolution’ wheat yields increased by the introduction of reduced height (*Rht*) genes. The resulting dwarf and semi-dwarf varieties benefit from improved resource allocation from the stem to the spike and reduced lodging, allowing more intensive nitrogen application (Hedden, 2003). Gibberellin (GA)-insensitive *Rht* genes (*Rht-A1*, *Rht-B1*, and *Rht-D1*) were shown to limit cell wall extensibility which decreases growth rates (Keyes *et al.*, 1989) without affecting development (Youssefian *et al.*, 1992). Moreover, the allele *Rht-B1c* (Wu *et al.*, 2011) and the GA-sensitive *Rht12* dwarfing gene (Chen *et al.*, 2013) delay heading.

The main abiotic factors affecting the timing of floral initiation and flowering are temperature and photoperiod, with temperature affecting both vernalization and general rate of development (Slafer *et al.*, 2015). These developmental transitions are controlled by major genes involved in the flowering pathway, namely vernalization (*Vrn*), photoperiod (*Ppd*), and earliness *per se* (*Eps*) genes (Slafer *et al.*, 2015). The *Ppd* and *Vrn* genes define photoperiod and vernalization requirements which jointly enable the transition to generative development and define time to flowering. On the other hand, *Eps* genes fine-tune the timing of floral transition and flowering, after vernalization and photoperiod requirements are fulfilled (Zikhali and Griffiths, 2015). While vernalization and photoperiod response are well known, the role of temperature *per se* remains less clear. Temperature affects all developmental phases, and warmer ambient temperatures generally accelerate growth and development in crops (Slafer and Rawson, 1994, 1995*a*,*c*; Atkinson and Porter, 1996; Fischer, 2011; Slafer *et al.*, 2015). However, it is unclear if temperature response governs growth rate and development independently. If so, the question remains as to whether there is enough genetic variability in temperature response to be used in a breeding context (Parent and Tardieu, 2012).

Genotypic variation for growth response to temperature was reported for wheat leaf elongation rate (Nagelmüller *et al.*, 2016), as well as for canopy cover growth (Grieder *et al.*, 2015). Kiss *et al.* (2017) reported significant genotype×temperature interactions in the timing of SE as well as temperature-dependent differences in the expression of *Vrn* and *Ppd* genes under controlled conditions. Under field conditions, the response of stem elongation to temperature has not yet been investigated at high temporal resolution.

In recent years, new high-throughput phenotyping technologies have enabled the monitoring of plant height with high accuracy and frequency in the field (Bendig *et al.*, 2013; Friedli *et al.*, 2016; Holman *et al.*, 2016; Aasen and Bareth, 2018; Hund *et al.*, 2019). We have previously demonstrated that the ETH field phenotyping platform (FIP; Kirchgessner *et al.*, 2016) can be used to accurately track the development of canopy height in a large set of wheat genotypes using terrestrial laser scanning (Kronenberg *et al.*, 2017). Considerable genotypic variation was detected for the start and end of SE which correlated positively with final canopy height (Kronenberg *et al.*, 2017).

While many temperature-independent factors affecting plant height are known, the influences of temperature-dependent elongation and timing of the elongation phase is less clear. We hypothesize that apart from temperature-independent factors, there is a genotype-specific response to ambient temperature which affects growth as well as the timing of developmental stages. To address this, we aimed to dissect the process towards final height into the following components: (i) temperature-independent elongation; (ii) temperature-dependent elongation; and (iii) the duration of the elongation phase determined by the start and end of the process. To achieve this, we present a method to assess and measure these three processes under field conditions by means of high-frequency, high-throughput phenotyping of canopy height development. The resulting data were combined with genetic markers to identify quantitative trait loci (QTLs) controlling the aforementioned processes.

## Materials and methods

### Experimental set-up, phenotyping procedures, and extracted traits

Field experiments were conducted in the FIP at the ETH research station in Lindau-Eschikon, Switzerland (47.449°N, 8.682°E, 520 m a.s.l.; soil type: eutric cambisol). We used a set of ~330 winter wheat genotypes (335–352 depending on the experiment) comprising current European elite cultivars (GABI wheat; Kollers *et al.*, 2013), supplemented with 30 Swiss varieties. These were monitored over three growing seasons in 2015, 2016, and 2017. Briefly, the field experiments were conducted in an augmented design with two replications per genotype using micro plots with a size of 1.4×1.1 m. In the 2017 growing season, the experiment was repeated again, with minor changes in genotypic composition. This resulted in 328 genotypes present across all three experiments. Details about the experimental set-up for the growing seasons 2015 and 2016 are described in Kronenberg *et al.* (2017).

Canopy height was measured twice weekly from the beginning of shooting (BBCH 31) using a light detection and ranging (LIDAR) scanner (FARO R Focus3D S 120; Faro Technologies Inc., Lake Mary, USA) mounted on the FIP (Kirchgessner *et al.*, 2016). Height measurements were concluded when no further height increase was observed in any of the genotypes. Canopy height data were extracted from the LIDAR data as described in Kronenberg *et al.* (2017).

The start, end, and duration of SE as well as final canopy height (FH) were extracted from the height data following Kronenberg *et al.* (2017): FH was defined as the point after which no increase in height was observed in several consecutive measurements. Normalized canopy height was calculated as the percentage of FH on each day of measurement for every plot and then linearly interpolated between measurement points. Growing degree-days after sowing until 15% FH (GDD_15_) and 95% FH (GDD_95_) were used as proxy traits for the start and end of SE, respectively. SE duration was recorded in thermal time (GDD_SE_) as well as in calendar days (time_SE_), as the difference between GDD_95_ and GDD_15_ (Kronenberg *et al.*, 2017). Heading date was recorded as GDD (heading_GDD_) when 50% of the spikes were fully emerged from the flag leaf sheath (BBCH 59; Lancashire *et al.*, 1991). Heading data for 2015 could not be evaluated due to insufficient data availability. Therefore, a third year of heading data was gathered in 2018, when the experiment was repeated again as described in Anderegg *et al.* (2020).

In order to investigate short-term growth response to temperature, average daily stem elongation rates (SERs) were calculated for each plot as the difference (∆) in canopy height (CH) between consecultive time points (*t*):

$$\begin{array}{l} SER=\Delta CH\;/\;\Delta t \end{array}$$ (1)

### Extracting growth response to temperature

**Fig. 1.**
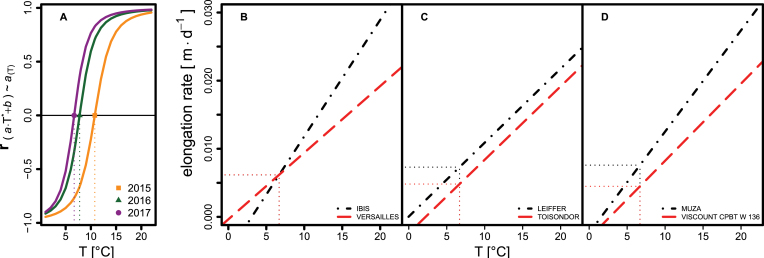
Illustration and interpretation for the parameters of the applied temperature response model (Equation 2). (A) Distribution of Pearson correlation coefficients between the intercept and slope of the linear model for individual years, depending on the temperature at which the intercept is calculated. Dotted vertical lines indicate the critical temperature (*T*_crit_) for individual years used to calculate the intercept. (B–D) Illustration of the relationship between intercept and slope on contrasting genotypes (dashed and dash-dotted lines). (B) The same vigour but differing in temperature response. (C) Both have the same temperature response but differ in vigour. (D) Genotypes differ in vigour as well as in temperature response. Horizontal and vertical dotted lines indicate vigour and *T*_crit_, respectively. The two contrasting genotypes per example (B–D) were selected from the 2017 data based on their vales for slope and intercept.

Temperature response was modelled by regressing average daily SER against average temperature of the respective interval for each plot within the respective year following

$$\begin{array}{l} SER=\left(a\;\times \;T\right)+{b_{T}}_{crit}+\varepsilon \end{array}$$ (2)

where *T* is the ambient temperature, *a* is the coefficient of the linear regression (i.e. growth response to ambient temperature; slp_SER~*T*_), and ε denotes the residual error. *b*_*T*crit_ is the model intercept at the temperature at which the correlation between intercept (int_SER~*T*_) and slope is zero (see below). Per definition, the intercept of a linear model would be calculated at *T*=0 °C—far outside the range of observed temperatures. In the observed data, the intercept at *T*=0 °C correlated strongly negatively with the slope (Fig. 1A) and thus did not add much additional information concerning the performance of the evaluated genotypes. Likewise, an intercept at 20 °C, at the upper range of the observed data, correlated strongly positively with the slope (Fig. 1A). Grieder *et al.* (2015) performed a similar analysis for the canopy cover development during winter and found a similar, strongly negative correlation between temperature response (slope) and growth at 0 °C (intercept). We sequentially calculated the intercept at 0.01 °C increments between 1 °C and 22 °C for each plot within a year. Subsequently, we calculated the Pearson correlation coefficient between the common slope of these models and each of the different intercepts (Fig. 1A). Based on this sequence, we empirically determined the critical temperature value (*T*_crit_) at which the correlation between slope and intercept was zero (Fig. 1A). Hence, *T*_crit_ is defined as the temperature at which intercept and slope are independent. Due to this independence, the value of the intercept at *T*_crit_ can be interpreted as the intrinsic growth component independent of temperature response herein referred to as ‘vigour’. Following this, two genotypes can show the same vigour but differ markedly in temperature response (Fig. 1B), have the same temperature response but differ in vigour (Fig. 1C), or differ for both temperature response and vigour (Fig. 1D).

### Statistical analysis

The data were processed stepwise as follows: (i) correction for design factors and spatial trends; (ii) application of linear models to each plot to determine growth response to temperature; and (iii) prediction of adjusted means and calculation of the heritability for all traits across years.

The spatial correction of canopy height per measurement time point was done using the R package SpATS (Rodríguez-Álvarez *et al.*, 2018) following:

$$\begin{array}{l} {Y_{i}}_{,j,k}=f\left(r,c\right)+G_{i}+R_{j}+C_{k} \end{array}$$ (3)

where *Y* is the phenotypic value for the a plot in the *j*th row in the *k*th column planted with the *i*th genotype, *f*(*r*,*c*) is a smoothed bivariate surface defined over rows and ranges of a virtual grid, *G*_*i*_ is the effect of the *i*th genotype (*i*=1, …, *n*; *n*=335–352 depending on the year), *R*_*j*_ is the effect of the *j*th row in the virtual grid, and *C*_*k*_ is the effect of the *k*th range. With the number of genotypes, the number of ranges/rows varied across years (17/21 in 2015, 18/20 in 2016, and 18/21 in 2017, respectively). The replications were arranged diagonally in this grid with a gap of five rows and ranges between them. Thus, for example in 2017, replication 1 was at rows 1–21 and ranges 1–18; replication 2 was at rows 24–41 and ranges 27–47 of the virtual grid.

The function *f*(*r*,*c*) describing the bivariate surface can be decomposed in a nested-type ANOVA structure as described by Rodríguez-Álvarez *et al*. (2018). The number of spline points was set to two-thirds of the total number of rows and ranges in the virtual grid, respectively.

From this model, the predicted genotypic best linear unbiased estimates (BLUEs) plus residual error were kept as spatially corrected plot values. Thus, these new plot values were corrected for spatial effect as well as for the random row and range effects, and used for the subsequent dynamic model. For a visualization of the applied SpATS correction, see Supplementary Fig. S1 at *JXB* online.

Genotypic BLUEs across the three seasons were calculated for all traits using the R package asreml-4 (Butler, 2018) following:

$$\begin{array}{l} y_{pij}=\mu +G_{i}+Y_{j}+GY_{ij}+\varepsilon_{pij} \end{array}$$ (4)

where *y*_*pij*_ is the spatialy corrected plot value of the respective trait (FH, GDD_15_, GDD_95_, GDD_SE_, time_SE_, int_SER~*T*_, or slp_SER~*T*_), μ is the overall mean, *G*_*i*_ the fixed effect of the genotypes common in all three years (*i*=1,…,328), *Y*_*j*_ is the fixed effect of the year (*j*=2015,…,2017), *GY*_*ij*_ is the random genotype-by-year interaction, and ε _*pij*_ is the residual error.

In order to estimate best linear unbiased predictors (BLUPs) and heritability (*H*^2^) across years, *G*_*i*_ in Equation 3 was set as a random term and heritability was calculated following (Falconer and Mackay, 1996) using:

$$\begin{array}{l} H^{2}=\frac{\sigma_{G}^{2}}{\sigma_{G}^{2}+\frac{\sigma_{GY}^{2}}{3}+\frac{\sigma_{\varepsilon }^{2}}{6}} \end{array}$$ (5)

where *H*^2^ is the broad sense heritability, σ ^2^_*G*_ is the genotypic variance, σ ^2^_*GY*_ is the genotype×year interaction variance, and σ ^2^_*ε*_ is the residual variance. For heading data, only one replicate per year was available. Plot-corrected values were extracted using SpATS (Rodríguez-Álvarez *et al.*, 2018), and heritability across 3 years was calculated by omitting the *GY* term in Equation 4 and dividing the residual variance by three, based on the three available year–site replications.

Genotypic BLUEs across 3 years were used for subsequent correlation analysis and genome-wide association study (GWAS). All statistical analyses were performed in the R environment (R Core Team, 2015).In order to investigate the relationship between FH, temperature response, and vigour, and to test for confounding *Rht* or *Ppd* effects on temperature response, FH was modelled using the linear model

$$\begin{array}{l} y_{i}=\;\;\;\mu +\underset{k=1}{\overset{6}{\sum }}\;\beta_{k}x_{k,i}+\underset{k=1}{\overset{5}{\sum }}\;\underset{m=k+1}{\overset{6}{\sum }}\;\beta_{k,m}x_{k,i}x_{m,i}+\varepsilon_{i} \end{array}$$ (6)

where *y*_*i*_ is FH of the *i*th genotype, µ is the model intercept, β _1–6_ are the main effect estimates of *x*=slp_SER~*T*_, int_SER~*T*_, GDD_SE_, *Rht-B1*, *Rht-D1*, or *Ppd-D1*, respectively. β _1,2_–β _5,6_ are all two-way interaction effects (*n*=15) and ε _i_ is the residual error. Genotypic data for *Rht-B1*, *Rht-D1*, and *Ppd-D1* alleles were available for 301 genotypes obtained from Kollers *et al.* (2014). There, genotyping of the *Rht-1* alleles was performed using PCR markers (Ellis *et al.*, 2002), while *Ppd-D1* alleles were genotyped by the presence or absence of a 2 kb insertion using specific primers (Beales *et al.*, 2007; Kollers *et al.*, 2014)

### Association study

GWAS was performed on the different traits to compare the phenotypic correlations with the underlying genetic architecture of the traits. As a positive control, FH data from Germany and France reported by Zanke *et al.* (2014b) were also compared and analysed.

Genotyping data were made previously by the GABI wheat consortium represented by the Leibniz Institute of Plant Genetics and Crop Plant Research (IPK; Zanke *et al.*, 2014a) using the 90K illumina SNP-chip (Cavanagh *et al.*, 2013; Wang *et al.*, 2014). Monomorphic single nucleotide polymorphisms (SNPs) were discarded. The remaining markers were mapped to the IWGSC reference genome (International Wheat Genome Sequencing Consortium, 2018) by BLASTN search using an E-value threshold <1e^–30^. The genome position with the lowest E-value was assigned as the respective marker location. Markers that could not be unequivocally positioned were dropped. After filtering SNPs with a minor allele frequency and missing genotype rate <0.05, a total of 13 450 SNP markers and 315 genotypes remained in the set. The reference genome position of *Rht*, *Ppd*, *Vrn*, and putative *Eps* genes was determined with BLASTN search as described above using published GenBank sequences (Supplementary Table S1).

To mitigate against multiple testing, relatedness, and population structure, three different methods were used to calculate marker–trait associations (MTAs) between phenotypic BLUPs and SNP markers. (i) We used a mixed linear model (MLM) including principal components among marker alleles as fixed effects and kinship as random effect to account for population structure (Zhang *et al.*, 2010). This approach was chosen to stringently prevent type I errors. The MLM GWAS was performed using the R Package GAPIT (v.2, Tang *et al.*, 2016). Kinship was estimated according to VanRaden (2008). (ii) In a generalized linear model (GLM) framework implemented in PLINK (Purcell *et al.*, 2007), association analysis was performed using SNP haplotype blocks consisting of adjacent SNP triplets. Using haplotype blocks takes the surrounding region of a given SNP into account, thus increasing the power to detect rare variants (Purcell *et al.*, 2007). (iii) Finally, the FarmCPU method (Liu *et al.*, 2016) was used, which is also implemented in GAPIT. FarmCPU tests individual markers with multiple associated markers as covariates in a fixed effect model. Associated markers are iteratively used in a random effect model to estimate kinship. Confounding between testing markers and kinship is thus removed while controlling type I error, leading to increased power (Liu *et al.*, 2016).

For all methods, a Bonferroni correction was applied to the pointwise significance threshold of α=0.05, to avoid false positives. Hence, only markers above –log_10_(*P*-value) >5.43 were considered significant.

Linkage disequilibrium (LD) among markers was estimated using the squared correlation coefficient (*r*^2^) calculated with the R package SNPrelate (Zheng *et al.*, 2012). A threshold of *r*^2^=0.2 (Gaut and Long, 2003) was applied to calculate the chromosome-specific distance threshold of LD decay. Putative candidate genes were identified by searching the IWGSC annotation of the reference genome (International Wheat Genome Sequencing Consortium, 2018) for genes associated with growth and development within the LD distance threshold around the respective MTA.

## Results

### Phenotypic results

**Fig. 2.**
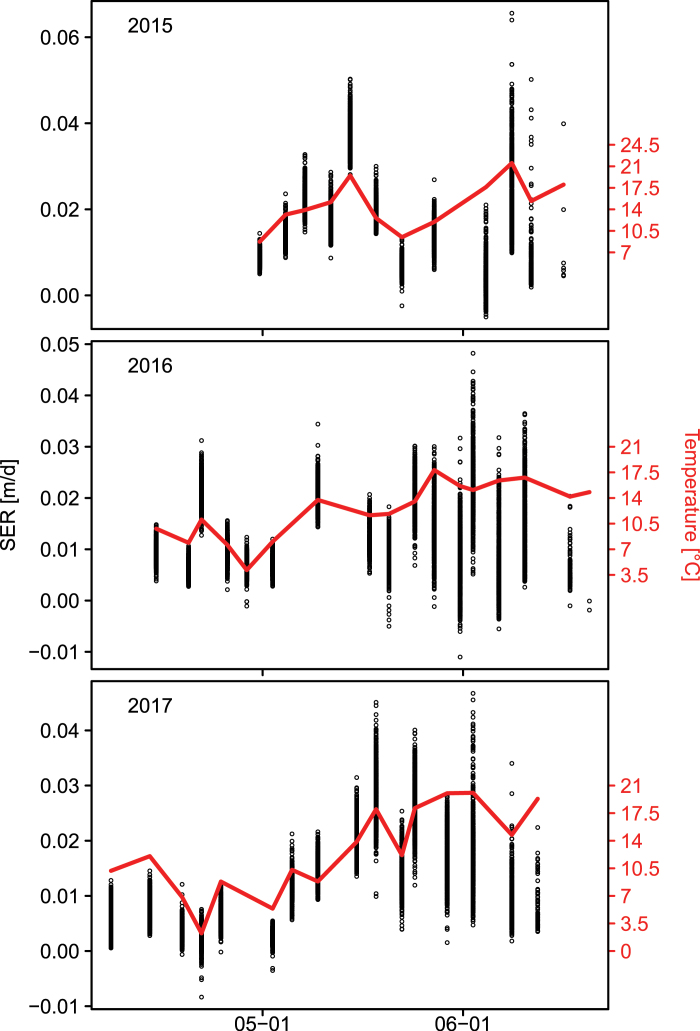
Relationship between the stem elongation rate (SER) and temperature. Plot-based SER raw data (*n*>700 per year) of >330 genotypes (black dots) as well as temperature (solid red line) are plotted against calendar time for the years 2015–2017.

**Fig. 3.**
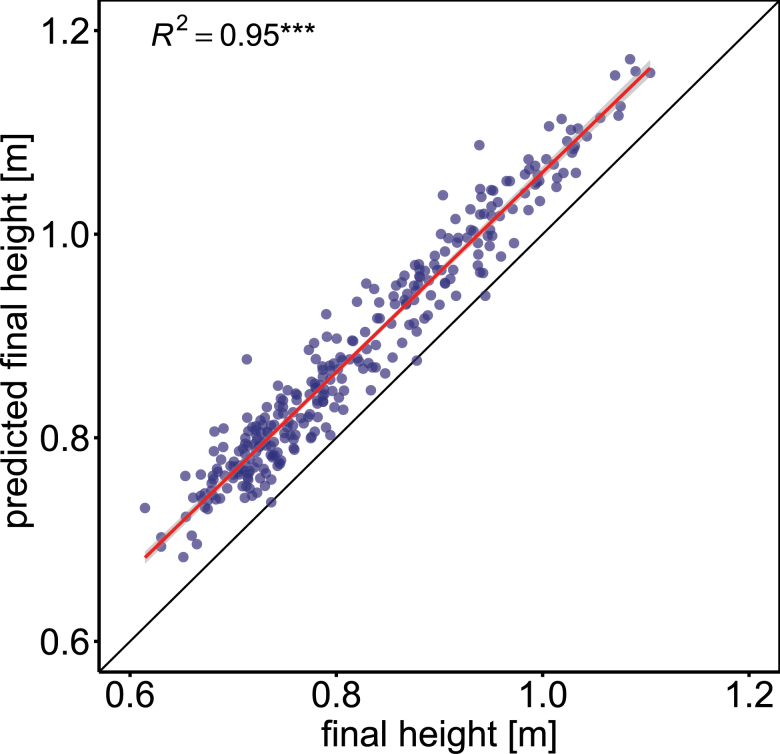
Prediction of final height based on temperature response (slp_SER~*T*_), vigour (int_SER~*T*_), and stem elongation duration (GDD_SE_). *Rht-B1*, *Rht-D1*, and *Ppd-D1* alleles and all two-way interactions were included to test for possible confounding of temperature response with final height or photoperiod (see Equation 6 and Table 2). The model was applied on the 3 year BLUEs of all genotypes with available allelic data of the respective genes (*n*=301).

We measured the canopy height of 710–756 plots per year, containing 335–352 wheat genotypes, for three consecutive years. In each season, measurements were made between 17 and 22 times during SE. Plot-based growth rates within single years extracted from these data indicate a clear relationship between growth and temperature for the period of SE, as depicted in Fig. 2. Towards the end of the measurement period in June, there was a larger deviation, which was also reflected in the quality of plot-based linear model fits of SER versus temperature (see Equation 2), summarized in Supplementary Fig. S2. For the 2015 and especially the 2016 experiment, *R*^2^ values were low and except for the 2017 experiment, the parameter estimates were not statistically significant (Supplementary Fig. S2a). Inspection of the best and worst model fits, however, shows that failure of fitting the model for single plots was levelled out by the replications within genotypes (Supplementary Fig. S2b). The weak model fits therefore did not affect the genotype ranking of adjusted means across replications. ANOVA revealed significant (*P*<0.001) genotypic effects for both slp_SER~T_ and int_SER~T_ across 3 years. Both traits showed high heritabilities across years (*H*^2^=0.81 for slp_SER~T_ and *H*^2^=0.77 for int_SER~T_; Table 1). Using the BLUEs of slp_SER~*T*_, int_SER~*T*_, and temperature sum for SE (GDD_SE_), FH could be predicted with high accuracy across different years (0.85≤*R*^2^≤0.89) by training a linear model on the BLUEs of one year and predicting it on the BLUEs of another independent year. In order to account for possible confounding *Rht* and *Ppd* effects, the allelic status of *Rht-B1*, *Rht-D1*, and *Ppd-D1* was included as contrasts in the model (Table 2). Of the 301 genotypes with available data, 7% and 58% carried the dwarfing alleles *Rht-B1b* and *Rht-D1b*, respectively, and 13% carried the photoperiod-insensitive allele *Ppd-D1a*. Training the model on the 3 year BLUEs resulted in a prediction accuracy of single years between *R*^2^=0.94 and *R*^2^=0.95 (Fig. 3). Type II ANOVA revealed significant effects for slp_SER~*T*_, int_SER~*T*_, GDD_SE_, *Rht-B1*, and *Rht-D1*. A significant (*P*<0.05) interaction effect was found between *Rht-D1* and *Ppd-D1*. Furthermore, weak interactions (*P*<0.1) were found for *Rht-B1:Ppd-D1*, int_SER*~T*_*:Rht-B1*, and int_SER~*T*_*:Rht-D1* (Table 2). High heritabilities across 3 years (0.54≤*H*^2^≤0.98; Table 1) were also found for the other traits: FH, start of SE, end of SE, SE duration, and heading. All traits showed moderate genotype×year interaction effects which were smaller (except for SE duration) than the genotypic effects across years (Table 1).

**Table 1.** Variance components and heritabilities for all investigated traits

Trait	Variance component	Estimate	SE	z ratio	% Total variance	Heritability
int_SER~*T*_	Gen_ID	7.024E-07	7.219E-08	9.730	49.01	0.77
	Gen_ID:Year	5.224E-07	3.491E-08	14.963	36.45	
	units!R	2.084E-07	8.920E-09	23.365	14.54	
slp_SER~*T*_	Gen_ID	7.348E-08	7.184E-09	10.229	55.00	0.81
	Gen_ID:Year	4.516E-08	2.925E-09	15.440	33.80	
	units!R	1.495E-08	6.396E-10	23.372	11.19	
FH	Gen_ID	1.226E-02	9.798E-04	12.511	92.24	0.98
	Gen_ID:Year	5.890E-04	4.568E-05	12.893	4.43	
	units!R	4.417E-04	1.890E-05	23.371	3.32	
GDD_15_	Gen_ID	1.226E+03	1.171E+02	10.471	56.64	0.82
	Gen_ID:Year	6.241E+02	4.370E+01	14.283	28.84	
	units!R	3.144E+02	1.345E+01	23.365	14.53	
GDD_95_	Gen_ID	1.190E+03	1.120E+02	10.624	56.84	0.84
	Gen_ID:Year	4.953E+02	3.958E+01	12.515	23.66	
	units!R	4.081E+02	1.747E+01	23.365	19.50	
time_SE_	Gen_ID	5.844E+00	8.095E-01	7.219	27.84	0.59
	Gen_ID:Year	9.481E+00	6.911E-01	13.717	45.16	
	units!R	5.668E+00	2.425E-01	23.370	27.00	
GDD_SE_	Gen_ID	5.665E+02	8.544E+01	6.631	24.14	0.54
	Gen_ID:Year	1.067E+03	8.007E+01	13.326	45.46	
	units!R	7.134E+02	3.052E+01	23.375	30.40	
heading_GDD_	Gen_ID	1.742E+03	1.481E+02	11.764	80.24	0.92
	units!R	4.290E+02	2.380E+01	18.028	19.76	

**Table 2.** Type II ANOVA table for the linear model^*a*^ used to predict final canopy height based on temperature response (slp_SER~*T*_), vigour (int_SER~*T*_), and stem elongation duration (GDD_SE_)

Predictor	Sum of squares	df	*F*-value	*P*r(>*F*)	
slp_SER~*T*_	7.63E-01	1	2.43E+03	5.08E-140	***
int_SER~*T*_	5.91E-01	1	1.88E+03	2.57E-126	***
GDD_SE_	1.29E-01	1	4.12E+02	5.74E-57	***
*Rht-B1*	3.16E-03	1	1.01E+01	1.69E-03	**
*Rht-D1*	3.53E-03	1	1.12E+01	9.13E-04	***
*Ppd-D1*	1.48E-06	1	4.71E-03	9.45E-01	
slp_SER~*T*_: int_SER~*T*_	2.56E-04	1	8.16E-01	3.67E-01	
slp_SER~*T*_:GDD_SE_	2.31E-05	1	7.36E-02	7.86E-01	
int_SER~*T*_:GDD_SE_	9.76E-06	1	3.11E-02	8.60E-01	
*Rht-B1*:*Ppd-D1*	8.94E-04	1	2.85E+00	9.26E-02	°
*Rht-D1*:*Ppd-D1*	1.42E-03	1	4.51E+00	3.45E-02	*
slp_SER~*T*_:*Rht-B1*	4.01E-06	1	1.28E-02	9.10E-01	
slp_SER~*T*_:*Rht-D1*	1.43E-06	1	4.56E-03	9.46E-01	
slp_SER~*T*_:*Ppd-D1*	5.53E-04	1	1.76E+00	1.85E-01	
int_SER~*T*_:*Rht-B1*	9.01E-04	1	2.87E+00	9.13E-02	°
int_SER~*T*_:*Rht-D1*	7.02E-04	1	2.23E+00	1.36E-01	
int_SER~*T*_:*Ppd-D1*	7.70E-04	1	2.45E+00	1.19E-01	
GDD_SE_:*Rht-B1*	1.71E-05	1	5.46E-02	8.15E-01	
GDD_SE_:*Rht-D1*	7.56E-04	1	2.41E+00	1.22E-01	
GDD_SE_:*Ppd-D1*	4.48E-05	1	1.43E-01	7.06E-01	
Residuals	8.79E-02	280			

*Rht-B1*, *Rht-D1*, band *Ppd-D1* alleles and all two-way interactions (the interaction effect of *Rht-B1* and *Rht-D1* was dropped due to singularity) were included to test for possible confounding of temperature response with final height or photoperiod. The model was applied on the 3 year BLUEs of all genotypes with available allelic data of the respective genes (*n*=301).

Asterisks and dots indicate the significance of the respective predictor (***P<0.001, **P<0.01, *P<0.05, °*P*<0.1).

^*a*^ See Equation 6.

### Phenology, temperature response, and final height were positively correlated

To evaluate the relationships between the traits measured, Pearson correlation coefficients were calculated for each trait pair. If not indicated otherwise, the reported correlations were highly significant (*P*<0.001)

**Fig. 4.**
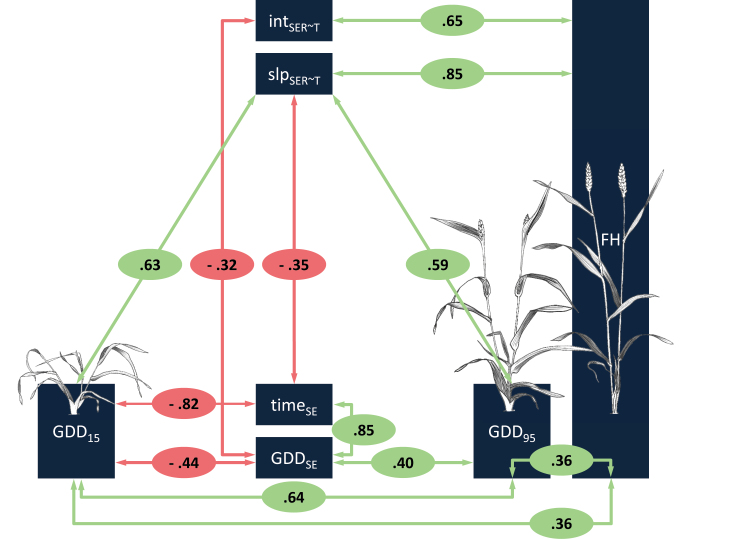
Key correlations among investigated traits. Pearson correlation coefficients between respective traits are given in red and green circles, where red denotes a negative correlation and green denotes a positive correlation. Weak correlations (*r*<0.3) are shown in the complete correlation matrix Supplementary Fig. S3. Illustrations of GDD_15_, GDD_95_, and FH were taken from Schürch *et al.* (2018).

Positive correlations were found among GDD_15_, GDD_95_, and FH (0.36≤*r*≤0.64, Fig. 4), indicating that taller genotypes were generally later in their development towards FH. Temperature response (slp_SER~*T*_) and vigour (int_SER~*T*_) also showed a strong, positive relationship to FH (*r*=0.85 and *r*=0.65, respectively). However, only temperature response correlated with GDD_15_ and GDD_95_ (*r*=0.63 and *r*=59, respectively), whereas vigour did not (*r*≤0.26, Supplementary Fig. S3).

As expected, SE duration in thermal time (GDD_SE_) was negatively correlated with GDD_15_ (*r*= –0.44) and positively correlated with GDD_95_ (*r*=0.4). However, GDD_SE_ did not correlate with FH (*r*= –0.01, *P*=0.874) or temperature response (*r*=0.006, *P*=0.285), although GDD_SE_ negatively correlated with vigour (*r*= –0.32). In contrast, SE duration in calendar days (time_SE_) was negatively correlated with temperature response (*r*= –0.35) and GDD_15_ (*r*= –0.82), indicating a longer SE phase for earlier genotypes. Heading showed strong positive correlations with GDD_15_ (*r*=0.61) and GDD_95_ (*r*=0.71), and a weak correlation to temperature response (*r*=0.29). Furthermore, heading correlated negatively with int_SER~*T*_ (*r*= –0.41) and showed no correlation to FH (*r*=0, *P*=0.934). Other weak correlations (*r*<0.3), that are not discussed, are shown in Supplementary Fig. S3.

### Linkage disequilibrium and population structure

Prior to MTA analysis, we evaluated population structure and LD. Principal component analysis (PCA) of the marker genotypes revealed no distinct substructure in the investigated population. The biplot of the first two principal components showed no apparent clusters, with the first component explaining 8% and the second component explaining 3.3% of the variation in the population (Supplementary Fig. S4). This is consistent with prior work using the same population (Kollers *et al.*, 2013; Yates *et al.*, 2019). On average across all chromosomes, LD decayed below an *r*^2^ of 0.2 at a distance of 9 Mb. There was, however, considerable variation in this threshold among the single chromosomes (Supplementary Table S2).

### Association study

**Fig. 5.**
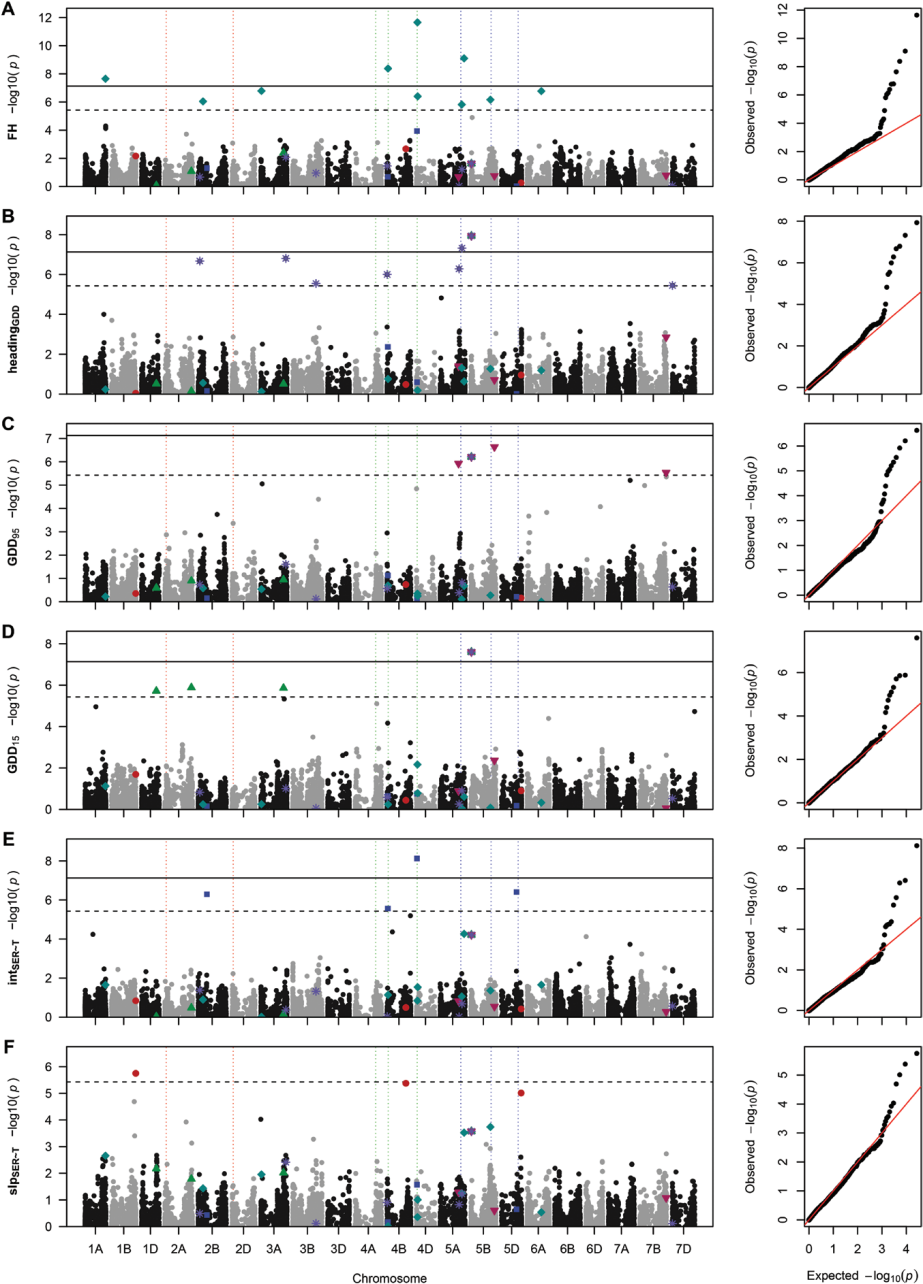
Manhattan plots and quantile–quantile plots depicting the GWAS results using FarmCPU for final height (FH, A), growing degree days until heading (heading_GDD_, B); end (GDD_95_, C) and start (GDD_15_, D) of stem elongation; vigour-related intercept (int_SER~*T*_, E); and temperature-related slope (slp_SER~*T*_, F) of stem elongation in response to temperature. Horizontal lines mark the Bonferroni-corrected significance threshold for *P*<0.05 (dashed line) and *P*<0.001 (solid line). Vertical dotted lines mark the positions of *Ppd-1* on chromosomes 2A and 2D (red), *Rht-1* on chromosomes 4A–4D (green), and *Vrn-1* on chromosomes 5A–5D. Significant marker–trait associations for slp_SER~*T*_ (red dots), int_SER~*T*_ (blue squares), GDD_15_ (green upright triangles), GDD_95_ (magenta downward-facing triangles), heading (violet asterisks), and FH (turquoise diamonds) are highlighted in all Manhattan plots.

Genome-wide association results differed markedly depending on the applied model. Using an MLM with kinship matrix and PCA as covariates resulted in no significant MTA for any trait (Supplementary Fig. S5). In contrast, the GLM using the haplotype method on temperature response yielded 2958 significant MTAs for α<0.05 and 1852 MTAs for α<0.001, respectively (Supplementary Fig. S6). However, investigation of the respective QQ-plots showed large *P*-value inflation in the haplotype method whereas the *P*-values were slightly deflated when using the MLM approach (Supplementary Figs S5, S6). In contrast, with FamCPU, the QQ-plots (Fig. 5) showed no *P*-value inflation, except for some markers. This pattern is expected, if population structure is appropriately controlled. Therefore, FarmCPU was chosen to be the most appropriate method for the given data, despite identifying fewer significant MTAs.

As a positive control, we compared our FH data and associated markers with data from Zanke *et al.* (2014b) who used the same population and SNP chip in field experiments in France and Germany. FH correlated strongly between the two studies (*r*=0.95), which is in accordance with the high heritability of the trait. In this study, we found 10 significant MTAs for FH (Table 3; Fig. 5). Zanke *et al.* (2014b) reported 280 significant MTAs for FH across several environments. Of these, only marker RAC875_rep_c105718_585 on chromosome 4D overlapped with the MTAs found in this study. However, by considering flanking markers, we found that of the remaining nine significant MTAs for FH, four were in LD with MTAs found by Zanke *et al.* (2014b; Supplementary Table S3). The significant MTA found for FH in this study are near known genes controlling FH. For example, Tdurum_contig64772_417 is 4 Mb upstream of *Rht*-B1 and RAC875_rep_c105718_585 is 7 Mb downstream of *Rht*-D1 on their respective group 4 chromosomes.

**Table 3.** Marker–trait associations for slp_SER~*T*_, int_SER~*T*_, GDD_15_, GDD_95_ heading_GDD_, and FH, including *P*-value, minor allele frequency (MAF), and allelic effect estimate

Trait	SNP	Chr	Position	*P*-value	MAF	Effect
slp_SER~*T*_	wsnp_Ex_c1597_3045682	1B	688 283 256	1.76E-06	0.19	–6.05E-05
	CAP7_c10839_300	4B	533 724 424	4.17E-06	0.24	–5.07E-05
	IAAV7104	5D	553 678 522	9.67E-06	0.13	–6.02E-05
int_SER~*T*_	RAC875_s109189_188	2B	248 149 774	5.08E-07	0.42	1.73E-04
	Ku_c63300_1309	4B	21 556 672	2.73E-06	0.10	–2.99E-04
	Kukri_rep_c68594_530	4D	12 773 259	7.47E-09	0.40	–2.30E-04
	Kukri_c6477_696	5D	423 502 809	3.89E-07	0.21	–2.03E-04
GDD_15_	wsnp_Ex_c12447_19847242	1D	416456386	1.91E-06	0.46	6.89E+00
	Tdurum_contig47508_250	2A	754 339 235	1.31E-06	0.21	9.41E+00
	Kukri_c55381_67	3A	648 868 234	1.38E-06	0.17	–1.00E+01
	Excalibur_c74858_243	5B	13 190 663	2.50E-08	0.47	–7.88E+00
GDD_95_	Excalibur_c49597_579	5A	521 934 666	1.19E-06	0.42	–6.58E+00
	Excalibur_c74858_243	5B	13 190 663	6.15E-07	0.47	–6.14E+00
	Tdurum_contig44115_561	5B	669 897 388	2.31E-07	0.13	–1.02E+01
	RAC875_c38693_319	7B	740 056 880	2.87E-06	0.20	7.51E+00
heading_GDD_	RAC875_c12766_461	2B	47 430 682	2.10E-07	0.39	–7.77E+00
	Kukri_rep_c106620_208	3A	714 300 397	1.55E-07	0.08	1.47E+01
	BS00022611_51	3B	659 787 924	2.83E-06	0.12	8.75E+00
	IAAV7221	4B	2 036 611	9.96E-07	0.07	–1.29E+01
	BS00000365_51	5A	538 000 573	5.19E-07	0.44	–7.65E+00
	IACX2540	5A	619 684 943	4.73E-08	0.35	9.39E+00
	Excalibur_c74858_243	5B	13 190 663	1.16E-08	0.47	–8.67E+00
	Excalibur_c46904_84	7D	5 198 912	3.55E-06	0.13	–9.46E+00
FH	Excalibur_c85499_232	1A	582 219 427	2.23E-08	0.11	2.52E-02
	wsnp_Ku_c11665_18999583	2B	139 070 721	9.07E-07	0.13	2.08E-02
	Kukri_c49280_230	3A	20 134 735	1.63E-07	0.08	2.90E-02
	Tdurum_contig64772_417	4B	26 491 482	4.18E-09	0.07	3.56E-02
	RAC875_rep_c105718_585	4D	25 989 162	2.20E-12	0.38	–2.56E-02
	BS00036421_51	4D	32 347 318	3.96E-07	0.37	–1.58E-02
	RAC875_c8231_1578	5A	613 588 253	1.52E-06	0.43	1.44E-02
	wsnp_Ku_rep_c71232_70948744	5A	679 663 586	7.93E-10	0.47	–2.21E-02
	BS00109560_51	5B	556 182 591	6.91E-07	0.46	–1.56E-02
	BS00022120_51	6A	396 301 470	1.68E-07	0.24	–2.01E-02

### Temperature response loci are independent of vigour loci

For slp_SER~*T*_, we detected one significant (LOD=5.75) MTA on chromosome 1B (wsnp_Ex_c1597_3045682) and two almost significant (LOD=5.38 LOD=5.01) MTAs on chromosomes 4B (CAP7_c10839_300) and 5D (IAAV7104), respectively (Fig. 5). All associated markers for slp_SER~*T*_ yielded small but significant allelic effects ranging from –0.061 mm °C^–1^ d^–1^ to –0.051 mm °C^–1^ d^–1^ (Table 3). The GWAS for int_SER~*T*_ yielded four significant MTAs on chromosomes 2B, 4B, 4D, and 5D, respectively (Table 3; Fig. 5). Start and end of SE yielded four MTAs each, and heading yielded eight MTAs (Table 3; Fig. 5).

Comparing the GWAS results for temperature response, vigour, FH, GDD_15_, GDD_95_, and heading revealed no common QTLs between slp_SER~*T*_ and any other trait. Only one marker (Excalibur_c74858_243) was significantly associated with both GDD_15_ and GDD_95_, as well as heading. The lack of overlap of MTAs between temperature response, vigour, and timing of critical stages indicates that they are genetically independent. However, there is a genetic connection between vigour and FH on the one hand and between the start and end of SE and heading on the other.

To identify potential causative genes underlying the QTLs, we searched the reference genome annotation around the respective QTL intervals. For temperature response, we found an increased presence of genes or gene homologues involved in the flowering pathway, namely *EARLY FLOWERING 3*, *FRIGIDA*, and *CONSTANS* (Table 4). Around the QTLs associated with vigour, the annotation showed genes associated with growth (i.e. *GRAS*, *CLAVATA*, *BSU1*, and *ARGONAUTE*) as well as developmental progress (i.e. *Tesmin/TSO1-like CXC domain*, *BEL1*, and *AGAMOUS*) (Table 5). Importantly, we found *GAI-like protein 1* 6 Mb upstream of marker Kukri_rep_c68594_530, which we identified as *Rht-D1* by blasting the *Rht*-D1 sequence (GeneBank ID AJ242531.1) against the annotated reference genome. Genes putatively underlying the QTLs for heading, GDD_15_, and GDD_95_ are listed in Supplementary Tables S4–S6. As expected, genes or gene homologues associated with the flowering pathway were found in the vicinity of the MTAs for heading. The common QTLs for heading, GDD_15_, and GDD_95_ on chromosome 5B (Excalibur_c74858_243) were found to be 6.6 Mb upstream of *FLOWERING LOCUS T* (Supplementary Tables S4–S6). Other flowering-associated genes found near the heading QTLs were *CONSTANS*, *FRIGIDA*, and a *FLOWERING LOCUS C*-associated gene (Supplementary Table S4). Moreover, a number of putative response regulators as well as genes putatively involved in light control of development (i.e. FAR1-RELATED SEQUENCE; Lin and Wang, 2004) were found near the heading QTLs (Supplementary Table S4). The remaining QTLs for GDD_95_ were near genes associated with developmental progress and flowering, such as *AGAMOUS*, *MEI2-like 1*, *HAPPLESS 2*, and *BEL1* (Supplementary Table S5). Genes near the remaining QTLs for GDD_15_ were associated with developmental progress (i.e. *FLOWERING LOCUS T*, *BEL1*, *TERMINAL EAR1-like*, and *FAR1-RELATED SEQUENCE*) as well as growth (i.e. *CLAVATA* and DELLA; Supplementary Table S6).

**Table 4.** Selected putative candidate genes for temperature response (slp_SER~*T*_) from the IWGSC reference genome annotation

Chr	SNP [Position]	r.start	r.end	Gene	Description	Distance
Chr1B	wsnp_Ex_c1597_3045682 [688 283 256]	688 282 509	688 286 431	TraesCS1B01G480600	Winged-helix DNA-binding transcription factor family protein	747
		688 352 414	688 354 696	TraesCS1B01G480700	HMG-Y-related protein A	–69 158
		687 710 716	687 719 885	TraesCS1B01G480100	Argonaute	572 540
		687 128 952	687 135 442	TraesCS1B01G479200	Zinc finger protein CONSTANS	1 154 304
		687 078 233	687 084 562	TraesCS1B01G479000	Zinc finger protein CONSTANS	1 205 023
		686 928 468	686 931 886	TraesCS1B01G478700	Zinc finger protein CONSTANS	1 354 788
		686 749 516	686 755 405	TraesCS1B01G478100	WD-repeat protein, putative	1 533 740
		685 645 287	685 649 392	TraesCS1B01G477400	Early flowering 3	2 637 969
Chr4B	CAP7_c10839_300 [533 724 424]	537 474 959	537 479 867	TraesCS4B01G266000	Protein FRIGIDA	–3 750 535
		541 363 317	541 365 139	TraesCS4B01G267700	Protein upstream of flc	–7 638 893
		542 582 729	542 583 265	TraesCS4B01G268300	MADS transcription factor	–8 858 305
Chr5D	IAAV7104 [553 678 522]	554 357 761	554 360 305	TraesCS5D01G544800	FRIGIDA-like protein, putative	–679 239
		554 467 487	554 472 596	TraesCS5D01G545100	Transducin/WD-like repeat- protein	–788 965
		556 226 523	556 234 480	TraesCS5D01G548800	Transducin/WD-like repeat- protein	–2 548 001

**Table 5.** Selected putative candiate genes for vigour (int_SER~*T*_) of temperature response from the IWGSC reference genome annotation

Chr	SNP [position]	r.start	r.end	Gene	Description	Distance
Chr2B	RAC875_s109189_188 [248 149 774]	243 569 388	243 571 100	TraesCS2B01G239400	GRAS transcription factor	4 580 386
Chr4B	Ku_c63300_1309 [21 556 672]	21 187 173	21 192 244	TraesCS4B01G028500	Tesmin/TSO1-like CXC domain- containing protein	369 499
		20 005 649	20 008 978	TraesCS4B01G026600	Argonaute family protein	1 551 023
		19 740 974	19 744 058	TraesCS4B01G026200	WD40 repeat-like protein	1 815 698
		23 404 428	23 408 188	TraesCS4B01G031300	BHLH family protein, putative, expressed	–1 847 756
		23 818 506	23 822 972	TraesCS4B01G032000	Protein UPSTREAM OF FLC	–2 261 834
		18 162 363	18 165 744	TraesCS4B01G025500	Homeobox protein BEL1 like	3 394 309
		18 091 908	18 093 975	TraesCS4B01G025400	BEL1-like homeodomain protein	3 464 764
		17 229 197	17 236 874	TraesCS4B01G024000	Argonaute protein	4 327 475
		17 017 132	17 019 148	TraesCS4B01G023300	AGAMOUS-like MADS-box transcription factor	4 539 540
		26 335 682	26 336 740	TraesCS4B01G036600	BRI1 suppressor 1 (BSU1)-like 3	–4 779 010
		26 824 399	26 827 490	TraesCS4B01G037200	WD-repeat protein, putative	–5 267 727
		15 427 017	15 431 870	TraesCS4B01G021500	Basic helix–loop–helix (bHLH) DNA-binding superfamily protein	6 129 655
		15 259 656	15 263 139	TraesCS4B01G021200	Basic helix–loop–helix (bHLH) DNA-binding superfamily protein	6 297 016
		15 146 117	15 150 854	TraesCS4B01G021100	Basic helix loop helix (BHLH) DNA-binding family protein	6 410 555
		14 710 395	14 711 057	TraesCS4B01G020800	Protein FAR1-RELATED SEQUENCE 5	6 846 277
		28 413 432	28 414 112	TraesCS4B01G041000	Sensitive to freezing 6	–6 856 760
		29 673 211	29 674 674	TraesCS4B01G042500	Fantastic four-like protein	–8 116 539
Chr4D	Kukri_rep_c68594_530 [12 773 259]	12 700 119	12 703 878	TraesCS4D01G028900	BHLH family protein, putative, expressed	73 140
		13 096 296	13 096 966	TraesCS4D01G029600	CLAVATA3/ESR (CLE)-related protein 25	–323 037
		13 196 859	13 200 535	TraesCS4D01G029700	Protein UPSTREAM OF FLC	–423 600
		11 364 404	11 369 466	TraesCS4D01G026100	Tesmin/TSO1-like CXC domain- containing protein	1 408 855
		10 746 363	10 750 251	TraesCS4D01G024300	Argonaute protein	2 026 896
		10 684 336	10 690 389	TraesCS4D01G024100	Argonaute family protein	2 088 923
		10 254 979	10 257 683	TraesCS4D01G023600	WD40 repeat-like protein	2 518 280
		15 768 990	15 772 059	TraesCS4D01G034500	WD-repeat protein, putative	–2 995 731
		9 495 616	9 501 619	TraesCS4D01G022600	Homeobox protein BEL1 like	3 277 643
		9 443 778	9 445 575	TraesCS4D01G022500	BEL1-like homeodomain protein 1	3 329 481
		9 069 403	9 071 423	TraesCS4D01G021100	MADS-box transcription factor	3 703 856
		16 584 271	16 584 948	TraesCS4D01G038400	Sensitive to freezing 6	–3 811 012
		8 777 205	8 779 670	TraesCS4D01G020300	Growth-regulating factor	3 996 054
		8 149 046	8 151 425	TraesCS4D01G019200	Basic helix–loop–helix (bHLH) DNA-binding superfamily protein	4 624 213
		8 135 666	8 137 454	TraesCS4D01G019100	Basic helix–loop–helix (bHLH) DNA-binding superfamily protein	4 637 593
		8 010 719	8 012 446	TraesCS4D01G018800	Basic helix–loop–helix (bHLH) DNA-binding superfamily protein	4 762 540
		7 992 104	7 995 445	TraesCS4D01G018700	Basic helix–loop–helix (bHLH) DNA-binding superfamily protein	4 781 155
		17 765 786	17 767 021	TraesCS4D01G039900	Fantastic four-like protein	–4 992 527
		18 781 062	18 782 933	TraesCS4D01G040400	GAI-like protein 1 (*Rht-D1*)	–6 007 803
		6 703 246	6 703 509	TraesCS4D01G015200	SAUR-like auxin-responsive protein family	6 070 013
		6 699 039	6 699 458	TraesCS4D01G015100	SAUR-like auxin-responsive protein family	6 074 220
		6 682 318	6 682 602	TraesCS4D01G015000	SAUR-like auxin-responsive protein family	6 090 941
		6 663 820	6 664 131	TraesCS4D01G014900	SAUR-like auxin-responsive protein family	6 109 439
		6 461 624	6 462 688	TraesCS4D01G013800	BRI1 suppressor 1 (BSU1)-like 3	6 311 635
		19 169 377	19 171 147	TraesCS4D01G040600	Protein FAR1-RELATED SEQUENCE 5	–6 396 118
		6 017 847	6 023 948	TraesCS4D01G012800	Protein FAR1-RELATED SEQUENCE 5	6 755 412
		4 128 933	4 133 919	TraesCS4D01G008400	WD-repeat protein, putative	8 644 326
		21 775 252	21 776 785	TraesCS4D01G046200	CONSTANS-like zinc finger protein	–9 001 993
Chr5D	Kukri_c6477_696 [423 502 809]	423 858 756	423 860 766	TraesCS5D01G334100	Armadillo repeat only	–355 947
		421 503 514	421 504 332	TraesCS5D01G329500	HVA22-like protein	1 999 295
		426 296 827	426 301 957	TraesCS5D01G337800	WD-repeat protein, putative	–2 794 018
		429 289 426	429 292 023	TraesCS5D01G341000	CONSTANS-like zinc finger protein	–5 786 617
		416 787 868	416 788 986	TraesCS5D01G325300	Protein Mei2	6 714 941
		416 625 946	416 628 639	TraesCS5D01G325200	Protein Mei2	6 876 863
		415 622 032	415 622 615	TraesCS5D01G323500	Auxin-responsive protein	7 880 777

### Vigour, temperature response, and the timing of SE affect final height

**Fig. 6.**
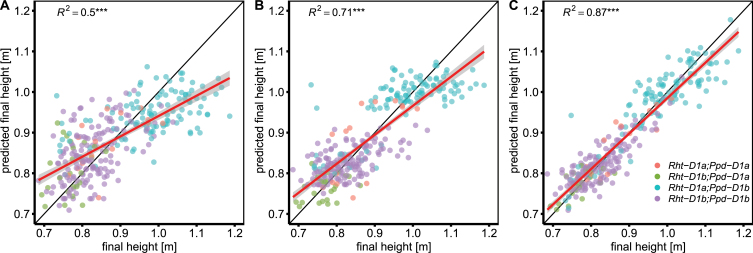
Prediction of final height based on QTLs and *Rht-B1*, *Rht-D1*, and *Ppd-D1* alleles. (A) The SNP alleles of significantly associated QTLs for temperature response (slp_SER~*T*_), vigour (int_SER~*T*_), and start (GDD_15_) and end (GDD_95_) of stem elongation were used in a linear model without considering interaction effects. (B) *Rht-B1*, *Rht-D1*, and *Ppd-D1* alleles were added to the model used in (A). (C) All two-way interaction effects among SNP alleles and *Rht-B1*, *Rht-D1*, and *Ppd-D1* alleles were included in the model. The models were applied to the 3 year BLUEs of all genotypes with available genotypic data (*n*=300) on all predictors. Colours indicate the allelic status regarding *Rht-D1* and *Ppd-D1* of the respective genotypes as depicted in (C).

The phenotypic correlations show a strong connection between temperature response, vigour, and FH as well as weaker connections between GDD_15_, GDD_95_, and FH. In order to examine this interdependency on a genetic level, we used a linear model to predict FH with the SNP alleles of the QTLs for slp_SER~*T*_, int_SER~*T*_, GDD_15_, and GDD_95_ as predictors. The model was able to predict FH with an accuracy *R*^2^=0.5; however, clusters in the data showed clear effects of *Rht-D1* and *Ppd-D1* alleles (Fig. 6A). Adding *Rht-B1*, *Rht-D1*, and *Ppd-D1* alleles as predictors increased the prediction accuracy to *R*^2^=0.71 (Fig. 6B). There were significant contributions by QTLs of all three traits; however, their effects were small compared with the obvious effects of *Rht-B1*, *Rht-D1*, and *Ppd-D1* (Table 6). Including all two-way interaction effects among the QTLs, *Rht-1* and *Ppd-1* increased the prediction accuracy to *R*^2^=0.87 (Fig. 6C), indicating a fine-tuning effect of temperature response, vigour, and timing of SE on FH.

**Table 6.** Type II analysis of variance for the prediction of final height using QTLs and *Rht-B1*, *Rht-D1*, and *Ppd-D1* alleles

Predictor [trait.QTL.chromosome]	SNP	Sum of squares	Df	*F*-value	*P*r(>*F*)	
slp_SER~*T*_.1.1B	wsnp_Ex_c1597_3045682	4.20E-03	1	1.08E+00	2.99E-01	
slp_SER~*T*_.2.4B	CAP7_c10839_300	7.97E-03	1	2.05E+00	1.53E-01	
slp_SER~T_.3.5D	IAAV7104	2.81E-02	1	7.23E+00	7.58E-03	**
int_SER~*T*_.1.2B	RAC875_s109189_188	1.72E-03	1	4.42E-01	5.07E-01	
int_SER~*T*_.2.4B	Ku_c63300_1309	1.64E-02	1	4.23E+00	4.07E-02	*
int_SER~*T*_.3.4D	Kukri_rep_c68594_530	7.47E-03	1	1.93E+00	1.66E-01	
int_SER~*T*_.4.5D	Kukri_c6477_696	6.06E-03	1	1.56E+00	2.13E-01	
GDD_15_.1.1D	wsnp_Ex_c12447_19847242	1.41E-02	1	3.64E+00	5.74E-02	°
GDD_15_.2.2A	Tdurum_contig47508_250	4.97E-02	1	1.28E+01	4.07E-04	***
GDD_15_.3.3A	Kukri_c55381_67	1.91E-02	1	4.91E+00	2.75E-02	*
GDD_15_.4|GDD_95_.2|heading.7.5B	Excalibur_c74858_243	7.64E-08	1	1.97E-05	9.96E-01	
GDD_95_.1.5A	Excalibur_c49597_579	4.04E-02	1	1.04E+01	1.40E-03	**
GDD_95_.3.5B	Tdurum_contig44115_561	1.03E-03	1	2.65E-01	6.07E-01	
GDD_95_.4.7B	RAC875_c38693_319	4.28E-03	1	1.10E+00	2.95E-01	
*Rht-B1*		3.32E-01	1	8.55E+01	6.02E-18	***
*Rht-D1*		6.71E-01	1	1.73E+02	3.79E-31	***
*Ppd-D1*		5.87E-02	1	1.51E+01	1.26E-04	***
Residuals		1.09E+00	282			

SNP alleles of the QTLs for temperature response (slp_SER~*T*_), vigour (int_SER~*T*_), start (GDD_15_), and end (GDD_95_) of stem elongation as well as *Rht-B1*, *Rht-D1*, and *Ppd-D1* alleles were used as predictors in a linear model (FH=ΣQTLslp_SER~*T*_+ΣQTLint_SER~*T*_+ΣGDD_15_+ΣQTLGDD_95_+*Rht-B1*+*Rht-D1*+*Ppd-D1*) without interaction effects (see Fig. 6B). The model was applied to the 3 year BLUEs of all genotypes with available genotypic data (*n*=300) on all predictors.

Asterisks and dots indicate the significance of the respective predictor (***P<0.001, **P<0.01, *P<0.05, °*P*<0.1).

## Discussion

In this study, we present a method to measure temperature response during stem elongation of wheat using high-throughput phenotyping of canopy height in the field. We found a genotype-specific response of wheat to change in ambient temperature which was correlated with the timing of the developmental key stages. We decomposed this growth dynamic into a genotype-specific vigour component and temperature response component using regression models. We further related these parameters to plant height and the timing of developmental key stages.

Linear regression models were used to describe wheat growth response to temperature for leaf elongation (Nagelmüller *et al.*, 2016), canopy cover (Grieder *et al.*, 2015), as well as SER (Slafer and Rawson, 1995*a*). Others proposed the use of a more complex, Arrhenius type of peak function to account for decreasing growth rates at supra-optimal temperatures (Parent and Tardieu, 2012). However such models are mainly applicable when the temperatures experienced by the crop exceed the temperature optimum. Wheat has its temperature optimum at ~27 °C (Parent and Tardieu, 2012). Temperatures in the measured growth intervals during SE did not exceed 25 °C and, given the temporal resolution of the data, a simple linear model is justified (Parent *et al.*, 2019).

The results of the correlation analysis show a clear connection between FH and temperature response (slp_SER~*T*_) as well as between FH and vigour (int_SER~*T*_). This is consistent with our hypothesis that FH can be described as a function of temperature-independent growth processes and as a function of temperature response during SE. Importantly, among all components, the temperature response was a significant driver of FH and also had a strong influence on the timing. Temperature response delayed the beginning of SE, leading to a later start and end of the whole phase. This finding might appear counter-intuitive: given the assumption that plants develop faster under higher ambient temperatures, a more responsive genotype should develop faster compared with a less responsive one. Slafer and Rawson (1995*b*) reported an accelerated development towards floral transition with increasing temperatures up to 19 °C, whereas higher temperatures slowed development. In that respect, a more responsive genotype would experience a stronger delay of floral transition under warm temperatures.

In terms of their correlation to FH, the effects of the timing of start and end of SE are less distinct. FH was more a function of faster growth than of a longer duration of growth, especially since genotypes with a strong temperature response had a shorter duration of SE. However, the timing of the start and end of SE was linked with temperature response. Based on this result and the correlations, it would appear that temperature response influences FH directly as well as indirectly by mediating the start and end of SE. Surprisingly, we found no correlation between heading and FH despite the positive correlation of both traits with GDD_95_. A correlation between heading date and FH would therefore be expected. Previous studies reported pleiotropic effects between plant height and heading time (Griffiths *et al.*, 2010; Mo *et al.*, 2018).

The question of whether these trait correlations are due to pleiotropic effects will substantially impact the breeding strategy (Chen and Lübberstedt, 2010). If the relationship between phenology, FH, and temperature response were to be to a large degree pleiotropic, these traits could not be independently selected. Alternative explanations are linkage and population structure. The GABI wheat panel is made of wheat varieties from different regions of Europe. As the examined traits are major drivers of adaptation to the different regions of Europe, we anticipate a very strong selection for both temperature response and timing of critical stages. Even if there is no apparent population structure at neutral markers, there may be a strong population structure at selected loci with a strong effect on local adaptation. Our phenotypic results showed a significant interaction effect between *Ppd-D1* and *Rht-D1* on FH, indicating either a co-selection or a pleiotropic effect of *Ppd-D1*. Pleiotropic effects between height and flowering time are known for maize and rice. For example, the *DWARF8* gene of maize encoding a DELLA protein is associated with height and flowering time (Lawit *et al.*, 2010) and strongly associated with climate adaptation (Camus-Kulandaivelu *et al.*, 2006). The rice *GHD7* locus has a strong effect on number of days to heading, number of grains per panicle, plant height, and stem growth (Xue *et al.*, 2008). In wheat, the dwarf gene *Rht-12* was shown to have a delaying effect on heading (Worland *et al.*, 1994; Chen *et al.*, 2013) as well as an additive interaction effect with *Ppd-D1* on plant height (Chen *et al.*, 2018). Furthermore, it was shown that the tall *Rht-D1a* and the photoperiod-sensitive *Ppd-D1b* allele positively affect leaf area and spike length throughout SE (Guo *et al.*, 2018). To further examine the relationship among the different traits, we consider the following GWAS analysis using stringent correction of population structure.

The GWAS results indicate an independent genetic control of FH, temperature response, and the timing of critical stages, whereas vigour and FH as well as heading time, and start and end of SE appear to be partly linked. Yet, FH could be predicted with surprising accuracy using the QTLs for temperature response, vigour, and start and end of SE, which reflects the correlations found in the phenotypic data.

Previous studies investigating the control of developmental key stages in wheat with respect to temperature generally adopted the concept that after fulfilment of photoperiod and vernalization, *Eps* genes act as fine-tuning factors independent of environmental stimuli (Kamran *et al.*, 2014; Zikhali and Griffiths, 2015). Increasing temperature, apart from vernalization, is thought to generally quicken growth and development independent of the cultivar (Slafer and Rawson, 1995*b*; Porter and Gawith, 1999; Slafer *et al.*, 2015). A genotype-specific temperature effect on the duration of different phases was not considered (Takahashi and Yasuda, 1971; Slafer and Rawson 1995*c*). It was, however, reported that photoperiod effects vary depending on temperature (Slafer and Rawson, 1995*c*). Under long days, Hemming *et al.* (2012) reported faster development and fewer fertile florets under high compared with low temperatures. Temperature-dependent effects were also found for different *Eps* QTLs (Slafer and Rawson, 1995*c*; Gororo *et al.*, 2001). It has previously been suggested that *Eps* effects could be associated with interaction effects between genotype and temperature fluctuations (Slafer and Rawson, 1995*c*; van Beem *et al.*, 2005).

The mechanisms of ambient temperature sensing and of its effects on growth and development are not yet well understood (Sanchez-Bermejo and Balasubramanian, 2016). However, important findings regarding ambient temperature effects on flowering time as well as on hypocotyl elongation have come from *Arabidopsis thaliana* (Wigge, 2013). With respect to these two traits, Sanchez-Bermejo and Balasubramanian (2016) reported distinct genotypic differences in temperature sensitivity. According to their results, the flowering pathway genes *FRIGIDA* (*FRI*), *FLOWERING LOCUS C* (*FLC*), and *FLOWERING LOCUS T* (*FT*) are major candidate genes for ambient temperature-mediated differences in flowering time (Sanchez-Bermejo and Balasubramanian, 2016). In the present study, we found *FRI* homologues near two of the three QTLs for temperature response. *FRI* and *FLC* act as the main vernalization genes in *A. thaliana* (Johanson *et al.*, 2000; Amasino and Michaels, 2010). In wheat, these genes are not yet well described. However, *FLC* orthologues were found to act as flowering repressors regulated by vernalization in monocots (Sharma *et al.*, 2017).

The most promising candidate gene for temperature response found near the QTLs on chromosome 1B is *EARLY FLOWERING 3* (*ELF3*). In Arabidopsis, *ELF3* was found to be a core part of the circadian clock involved in ambient temperature response (Thines and Harmon, 2010). In barley, *ELF3* was shown to be involved in the control of temperature-dependent expression of flowering time genes (Ejaz and von Korff, 2017). A mutant *ELF3* accelerated floral development under high ambient temperatures while maintaining the number of seeds (Ejaz and von Korff, 2017). Furthermore, *ELF3* has been reported as a candidate gene for *Eps1* in *Triticum monococcum* (Alvarez *et al.*, 2016) as well as in wheat (Zikhali *et al.*, 2016). A recent study in wheat showed an interaction between *Eps-D1* and ambient temperature which corresponded to different expression of *ELF3* (Ochagavía *et al.*, 2019). In this study, we directly measured growth response to temperature during SE and found a significant MTA near *ELF3* on chromosome 1B. Following Ochagavía *et al.* (2019), this indicates that growth response to temperature is connected to *Eps-B1* which is a homologue to *Eps-D1*. Furthermore, the temperature×*Eps-D1* interaction effects on heading reported by Ochagavía *et al.* (2019) are in agreement with the correlations found among growth response to temperature, GDD_15_, GDD_95_, and heading in the present study.

One important aspect we could not address in this study is the interaction of genotype-specific temperature response with vernalization and photoperiod (Slafer and Rawson, 1995*c*; Gol *et al.*, 2017; Kiss *et al.*, 2017). Due to the climate conditions in Switzerland, we expect fulfilment of vernalization requirement in all genotypes. However, due to the broad geographic origin of the investigated genotypes, the relationship between temperature response and the timing of SE might be confounded by different photoperiod requirements. Nevertheless, the correlations between earliness and temperature response are in agreement with Ochagavia *et al.* (2019). It also remains unclear whether and to what extent temperature response varies across different developmental phases and how temperature response relates to other environmental stimuli such as vapour pressure deficit or radiation. Nevertheless, the results of this study present valuable information towards a better understanding of temperature response in wheat and may be of great importance for breeding. Temperature response could provide a breeding avenue for local adaptation as well as the control of plant height.

With the recent advancements in unmanned aerial vehicle (UAV)-based phenotyping techniques, the growth of canopy cover and canopy height can be measured using image segmentation and structure from motion approaches (Bareth *et al.*, 2016; Aasen and Bareth, 2018; Roth *et al.*, 2018). Thus, temperature response can be investigated during the development of the vegetative canopy cover (Grieder *et al.*, 2015) and during the generative height development as demonstrated here. It can also be assessed in indoor platforms (e.g. Parent and Tardieu, 2012) and the field using a leaf length tracker (Nagelmüller *et al.*, 2016) measuring short-term responses of leaf growth to diurnal changes in temperature. Combining this information may greatly improve our understanding about the genetic variation in growth response to temperature.

Together, the results of this study indicate that temperature response may be exploitable as a breeding trait to adjust phenology towards specific environments, through either phenotypic or marker-assisted selection. Furthermore, a better understanding of temperature response may enhance the capability of crop models to predict crop performance under future climate change scenarios.

### Conclusion and outlook

Modern phenotyping platforms hold great promise to map the genetic factors driving the response of developmental processes to environmental stimuli. To the best of our knowledge, this is the first experiment dissecting the SE process into its underlying components: temperature-dependent elongation, temperature-independent vigour, and duration of elongation. The independent loci detected for these traits suggest that it is possible to select them independently. The detected loci may be used to fine-tune height and the beginning and end of SE as they explain a substantial part of the overall genotypic variation. With increases in automation, growth processes may be monitored in the field on a daily basis or even multiple times per day. This will increase the precision in assessing genotype responses to the fluctuation in meteorological conditions and will allow quantification of the relationship of these responses to yield. Remote sensing by means of UAVs in combination with photogrammetric algorithms will allow measurement of these traits in breeding nurseries. We believe that this is paving the way for a more informed selection to climate adaptation within individual growing seasons.

## Supplementary data

The following supplementary data are available at *JXB* online.

Fig. S1. Correction of canopy height for spatial as well as random row and range effects.

Fig. S2. Summary of plot-based linear model fits of stem elongation rate versus temperature.

Fig. S3. Pearson correlation coefficients among 3 year BLUEs of all investigated traits.

Fig. S4. Principal component analysis among marker genotypes.

Fig. S5. Manhattan plots and quantile–quantile plots depicting the GWAS results using the MLM approach.

Fig. S6. Manhattan plots and quantile–quantile plots depicting the GWAS results using the GLM approach.

Table S1. Genes of interest related to floral transition and flowering.

Table S2. Chromosome-wise distance thresholds for LD decay <*r*^2^=0.2

Table S3. Corresponding marker–trait associations for final canopy height with respect to Zanke *et al*. (2016).

Table S4. Selected putative candidate genes for heading_GDD_ from the IWGSC reference genome annotation.

Table S5. Selected putative candidate genes for GDD_95_ from the IWGSC reference genome annotation.

Table S6. Selected putative candidate genes for GDD_15_ from the IWGSC reference genome annotation.

Table S7. Three-year BLUEs of the investigated traits FH, heading_GDD_, GDD_15_, GDD_95_, GDD_SE_, time_SE_, slp_SER~*T*_, and int_SER~*T*._

eraa471_suppl_Supplementary_File001

eraa471_suppl_Supplementary_File002

## Data Availability

Processed phenotypic data are available as Supplementary data. Unprocessed data and analysis scripts are available from the authors upon reasonable request.
