# Supplementary material for: Temperature response of wheat affects final height and the timing of stem elongation under field conditions
Source: J Exp Bot. 2020 Oct 15;72(2):700–17. doi: 10.1093/jxb/eraa471 (PMC7853599; doi:10.1093/jxb/eraa471)
Supplement: eraa471_suppl_Supplementary_File001 [file eraa471_suppl_supplementary_file001.pdf]

Supplementary Data

Temperature response of wheat affects final height and the timing of stem elongation under field conditions

Lukas Kronenberg[a], Steven Yates[b], Martin P. Boer[c], Norbert Kirchgessner[a], Achim Walter[a], Andreas Hund[a]

[a] Crop Science, Institute of Agricultural Sciences , ETH Zürich, 8092 Zurich, Switzerland
[b] Molecular Plant Breeding, Institute of Agricultural Sciences , ETH Zürich, 8092 Zurich, Switzerland
[c] Biometris, Wageningen University & Research, 6708 PB Wageningen, The Netherlands

Fig. S1: Correction of canopy height for spatial as well as random row and range effects.

Fig. S2: Summary of plot based linear model fits of stem elongation rate vs. temperature.

Fig. S3: Pearson correlation coefficients among 3-year BLUES of all investigated traits.

Fig. S4: Principal component analysis among marker genotypes.

Fig. S5: Manhattan plots and quantile-quantile plots depicting the GWAS results using the MLM approach.

Fig. S6: Manhattan plots and quantile-quantile plots depicting the GWAS results using the GLM approach.

Table S1: Genes of interest related to floral transition and flowering.

Table S2: Chromosome wise distance thresholds for LD-decay < r^2 = 0.2

Table S3: Corresponding marker-trait associations for final canopy height with respect to Zanke et al. 2016.

See supplementary file 2 for tables S4 – S7:

Table S4: Selected putative candidate genes for $heading_{GDD}$ from the IWGSC reference genome annotation.

Table S5: Selected putative candidate genes for GDD_{95} from the IWGSC reference genome annotation.

Table S6: Selected putative candidate genes for GDD_{15} from the IWGSC reference genome annotation.

Table S7: 3-year BUEs of the investigated traits FH, $heading_{GDD}$, GDD_{15}, GDD_{95}, GDD_{SE}, $time_{SE}$, $slp_{SER\sim T}$, $int_{SER\sim T.}$

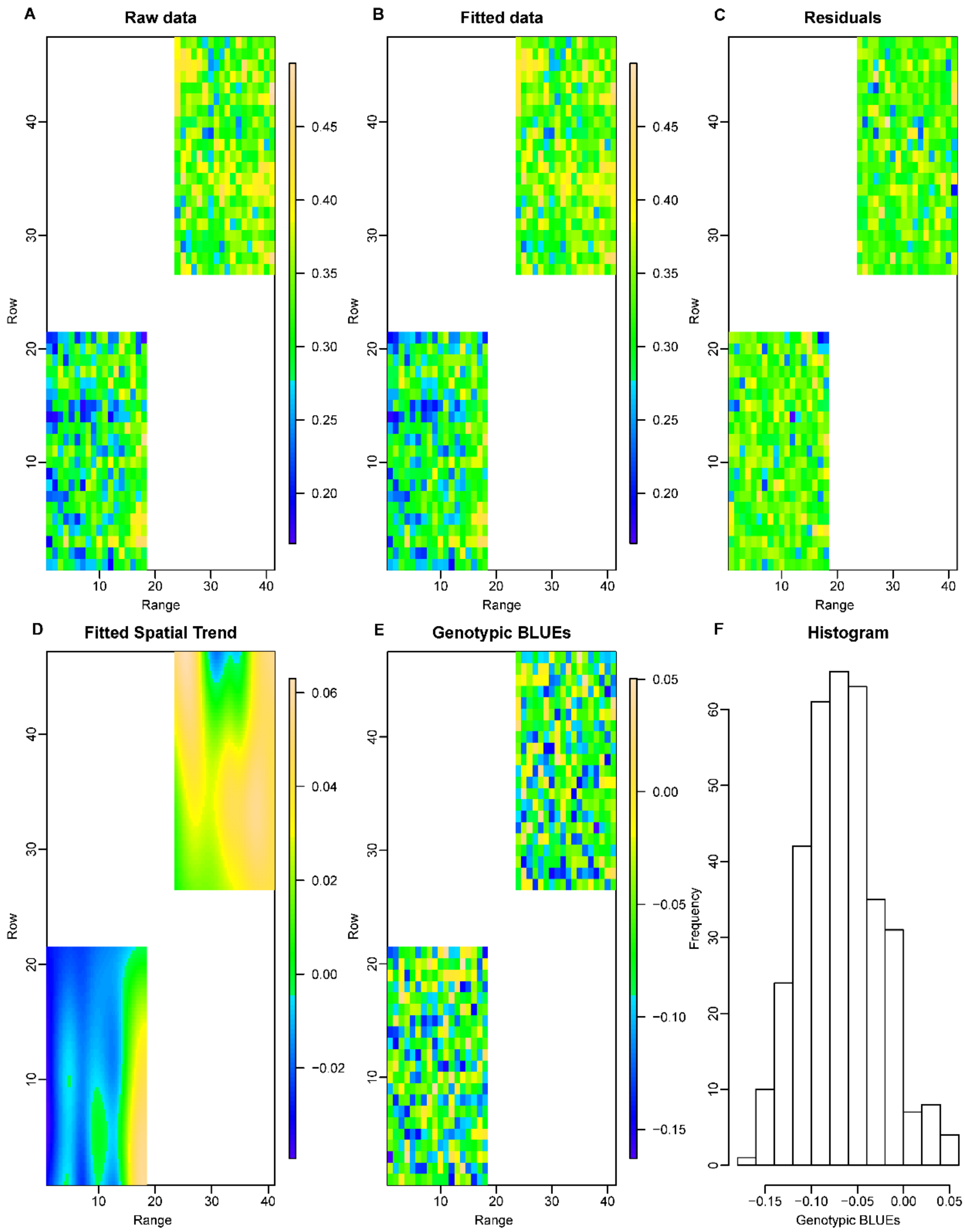

Fig. S1: Correction of canopy height for spatial as well as random row and range effects. The spatial correction of canopy height was done at each measurement time point by fitting a smoothed bivariate surface defined over rows and ranges to the raw canopy height data (see eq. 3 in Material and Methods) using the R-package SpATS (Rodríguez-Álvarez et al., 2018, Spatial Statistics). The figure shows the spatial correction for the time point 2017-05-09 displaying the raw canopy height data (A), the canopy height data fitted by the model (B), the residuals (C), the fitted spatial trend (D) as well as the genotypic estimates (E) and their distribution (F).

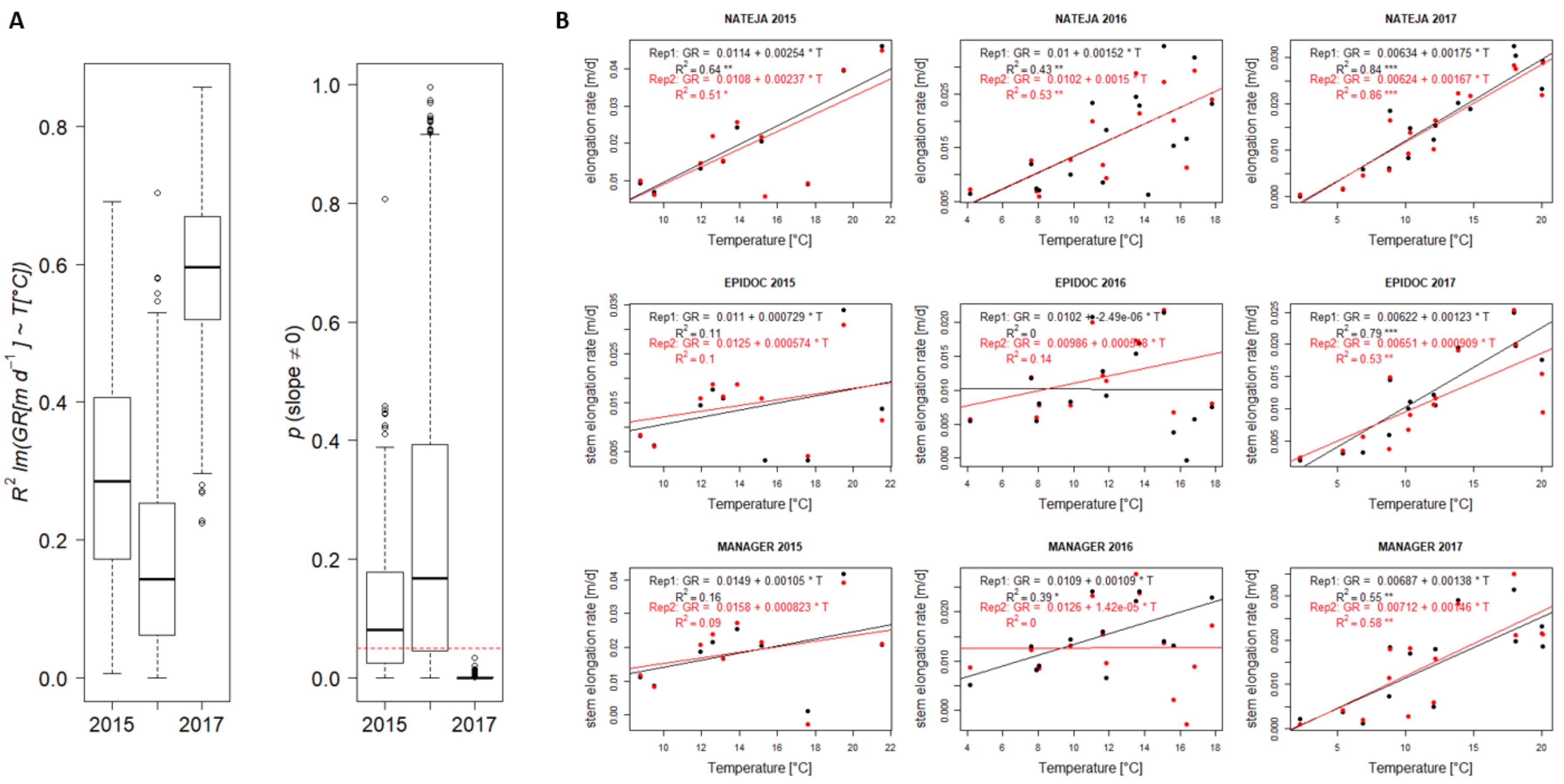

Fig. S2: Summary of plot based linear model fits of stem elongation rate vs. temperature. A Distribution of linear model R^2 and p-values of plot based linear model fits grouped by year. **B.** Genotype showing the best (NATEJA 2017 Rep1) and the two genotypes showing worst (EPIDOC 2016 Rep1; MANAGER 2016 Rep2) plot based linear model fit out of all plot based linear models fitted.

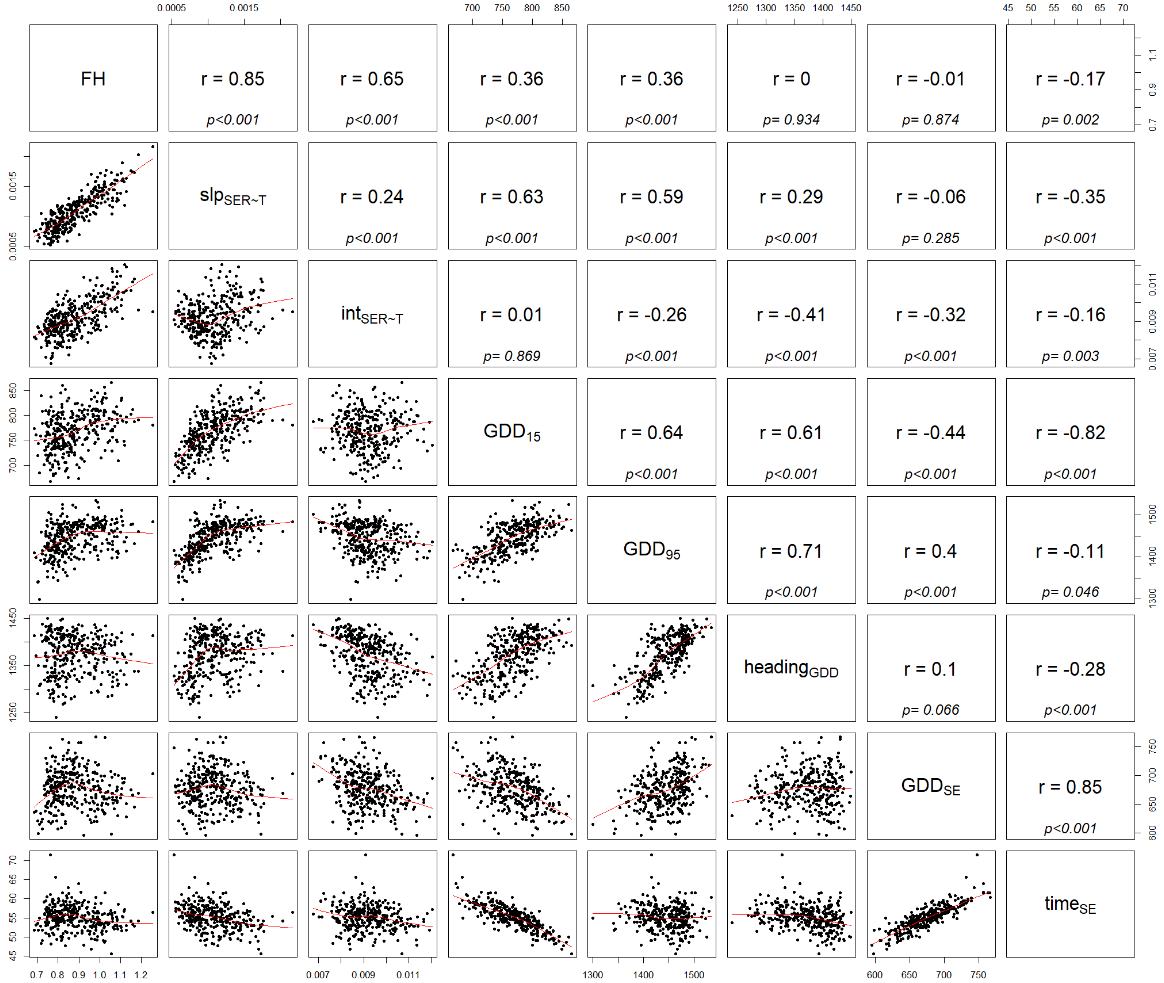

Fig. S3: Pearson correlation coefficients among 3-year BLUEs of all investigated traits.

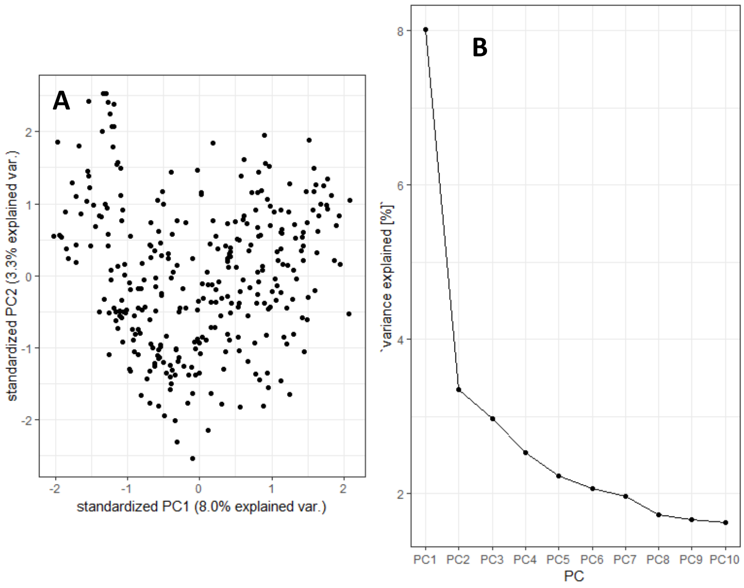

Fig. S4: Principal component analysis among marker genotypes. A: Biplot of the first two principal components. B: Screeplot showing the explained proportion of the variance for individual components.

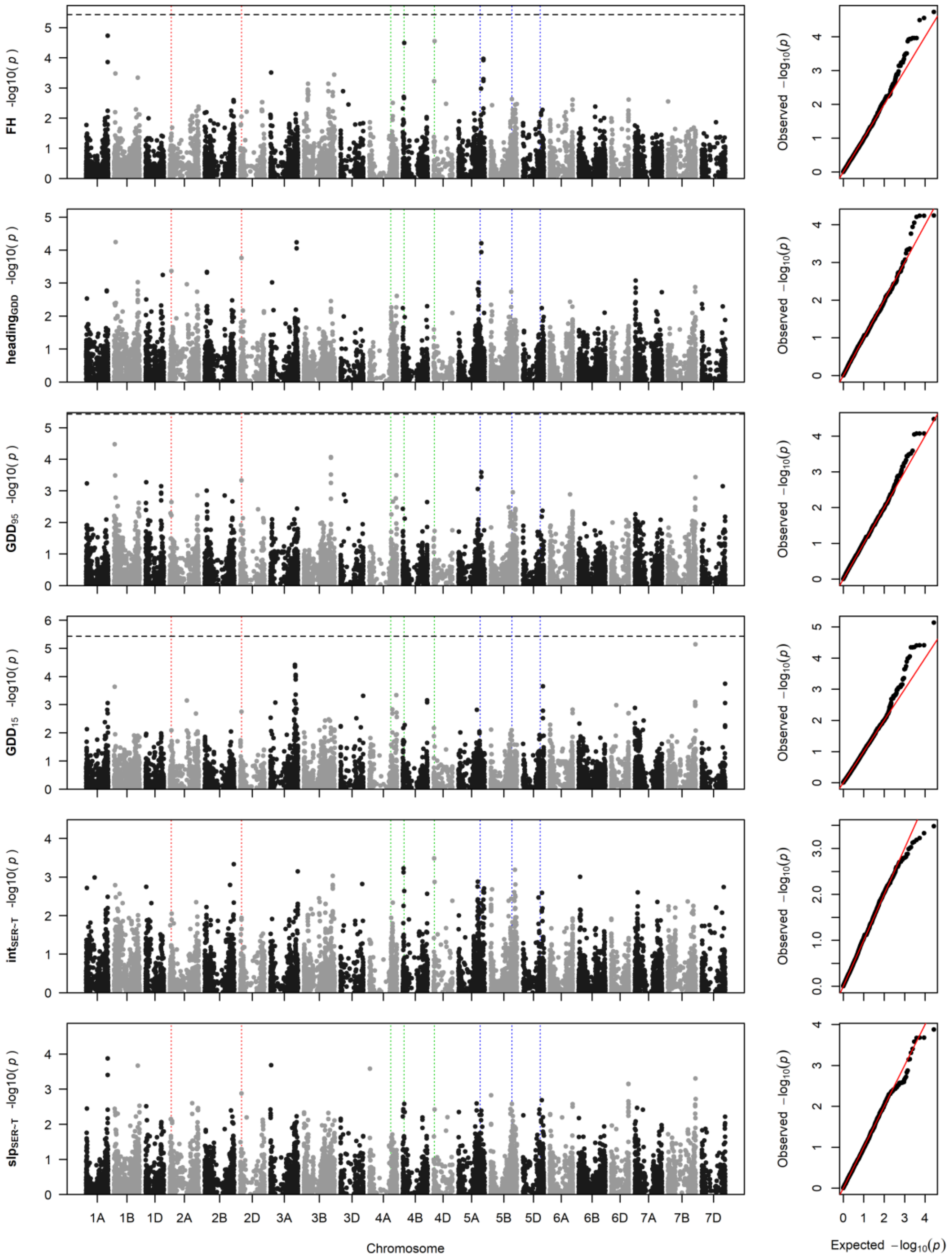

Fig. S5: Manhattan plots and quantile-quantile plots depicting the GWAS results using the MLM approach for final height (FH), heading in growing degree days ($heading_{GDD}$), GDD until start (GDD_{15}) and end (GDD_{95}) of stem elongation; vigour-related intercept ($int_{SER\sim T}$) and temperature-related slope ($slp_{SER\sim T}$) of stem elongation in response to temperature. Horizontal lines mark the Bonferroni corrected significance threshold for $p < 0.05$ (dashed line) and $p < 0.001$ (solid line). Vertical dotted lines mark the positions *Ppd-1* on chromosomes 2A and 2D (red), *Rht-1* on chromosomes 4A-4D (green) and *Vrn-1* on chromosomes 5A-5D.

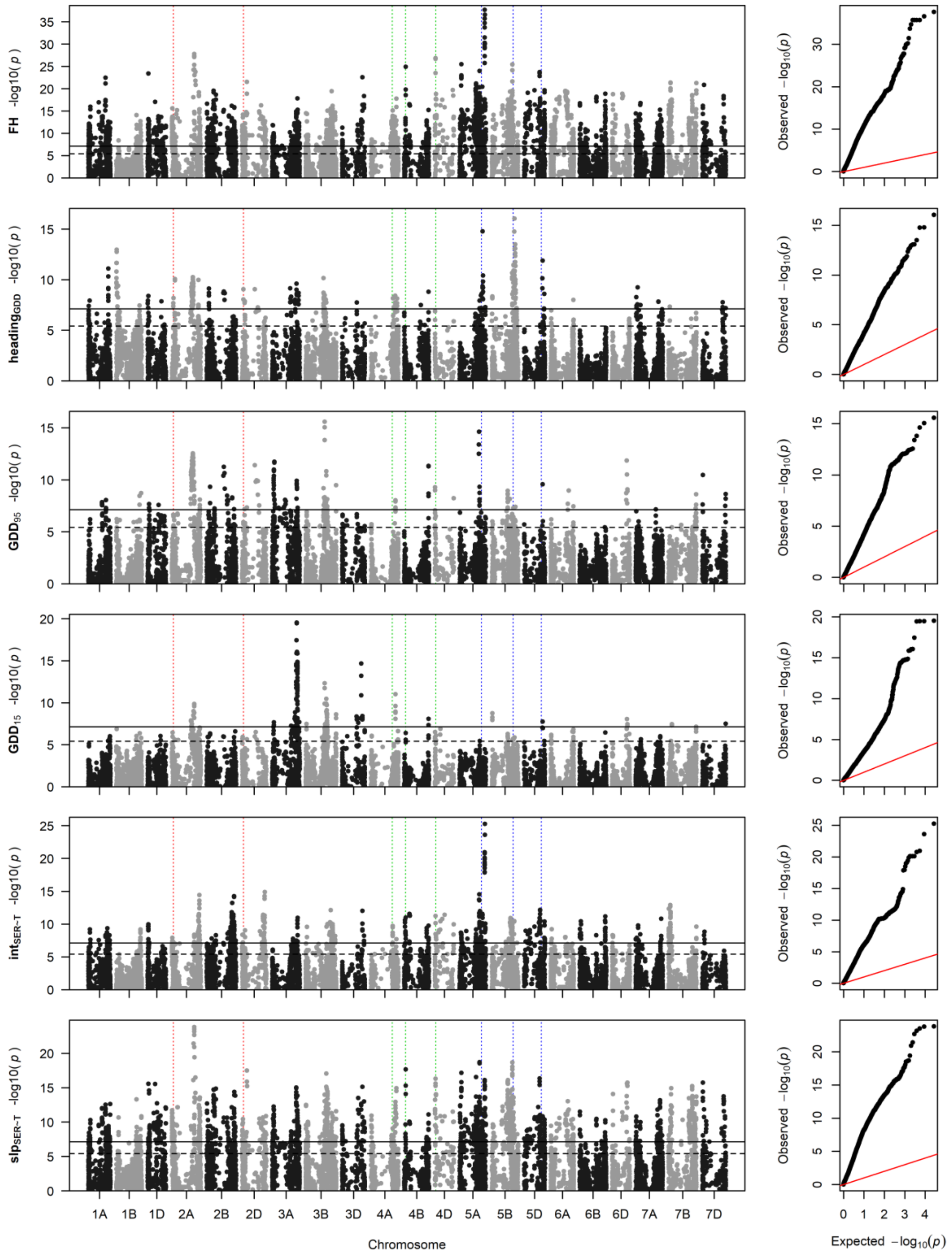

Fig. S6: Manhattan plots and quantile-quantile plots depicting the GWAS results using the GLM approach for final height (FH), heading in growing degree days ($heading_{GDD}$), GDD until start (GDD_{15}) and end (GDD_{95}) of stem elongation; vigour-related intercept ($int_{SER\sim T}$) and temperature-related slope ($slp_{SER\sim T}$) of stem elongation in response to temperature. Horizontal lines mark the Bonferroni corrected significance threshold for $p < 0.05$ (dashed line) and $p < 0.001$ (solid line). Vertical dotted lines mark the positions *Ppd-1* on chromosomes 2A and 2D (red), *Rht-1* on chromosomes 4A-4D (green) and *Vrn-1* on chromosomes 5A-5D.

Table S1: Genes of interest related to floral transition and flowering. Genome positions (r.start-r.end) were derived by blasting the respective published sequence (GenBank ID; https://www.ncbi.nlm.nih.gov/genbank/) against the IWGSC reference genome.

Gene	Chr	r.start	r.end	GenBank ID
Ppd-A1	chr2A	36'934'562	36'933'892	DQ885753.1
Ppd-D1	chr2D	33'953'359	33'952'698	DQ885766.1
Rht-A1	chr4A	582'479'578	582'477'716	JF930277.1
Rht-B1	chr4B	30'861'382	30'863'247	JX993610.1
Rht-D1	chr4D	18'781'062	18'782'933	AJ242531.1
TaELF3	chr1D	493'485'605	493'484'553	KR055809.1
TaFT3-A1	chr1A	528'066'476	528'066'282	KX161737.1
TaFT3-B1	chr1B	581'414'952	581'414'758	KX161739.1
TaFT3-D1	chr1D	430'469'335	430'469'144	KX161740.1
Vrn-A1	chr5A	587'423'240	587'423'056	AY616452.1
Vrn-B1	chr5B	573'815'903	573'815'719	AY747603.1
Vrn-B3	chr7B	9'703'464	9'703'735	DQ890162.1
Vrn-D1	chr5D	467'184'278	467'184'094	AY747597.1
Vrn-D4	chr5D	467'184'278	467'184'094	KR422424.1

Table S2 Chromosome wise distance thresholds for LD-decay < r^2 = 0.2

Chromosome	r^2 threshold [bp]
1A	6'161'631
1B	17'286'788
1D	12'822'505
2A	10'381'104
2B	11'361'169
2D	10'416'001
3A	9'887'297
3B	5'969'718
3D	4'233'196
4A	4'298'677
4B	11'488'268
4D	4'220'358
5A	10'344'140
5B	17'533'559
5D	4'862'712
6A	4'692'731
6B	19'679'489
6D	1'416'632
7A	7'133'812
7B	6'604'947
7D	NA

Table S3 Corresponding marker-trait associations for final canopy height with respect to Zanke et al. 2014*b*. MTA Zanke et al 2014*b* denotes marker trait associations reported by Zanke et al. 2014*b*, Closest MTA denotes the closest respective associated marker found in this study. Distance gives the distance in base pairs and r^2 the pairwise linkage disequilibrium between the two respective SNP.

Chr	MTA Zanke et al. 2014*b*	Closest MTA	distance	r^2
2B	Excalibur_c23185_155	wsnp_Ku_c11665_18999583	1'679'482	0.25
5A	wsnp_Ex_c23795_33033959	wsnp_Ku_rep_c71232_70948744	1'457	0.99
5A	Kukri_c75091_220	wsnp_Ku_rep_c71232_70948744	-5'660	0.99
5B	wsnp_Ex_c5155_9140608	BS00109560_51	2'442'796	0.82
5B	wsnp_Ex_c54092_57099525	BS00109560_51	-4'850'924	0.55
6A	BS00062823_51	BS00022120_51	51'119'779	0.60
